# Cross-sectional study of medical advertisements in a national general medical journal: evidence, cost, and safe use of advertised versus comparative drugs

**DOI:** 10.1186/s41073-021-00111-9

**Published:** 2021-05-10

**Authors:** Kim Boesen, Anders Lykkemark Simonsen, Karsten Juhl Jørgensen, Peter C. Gøtzsche

**Affiliations:** 1 Nordic Cochrane Centre, Rigshospitalet Dept. 7811, 2100 Copenhagen, Denmark; 2 Current address: Meta Research Innovation Center Berlin (METRIC-B), Berlin Institute of Health, Charité Universitätsmedizin, QUEST Center for Transforming Biomedical Research, Berlin, Germany; 3 Centre for Evidence-Based Medicine (CEBMO) and Cochrane Denmark, Dept. Clinical Research, University of Southern Denmark, Odense, Denmark; 4 Open Patient data Exploratory Network (OPEN), Odense University Hospital, Odense, Denmark; 5 Institute for Scientific Freedom, 2970 Copenhagen, Denmark

## Abstract

**Background:**

Healthcare professionals are exposed to advertisements for prescription drugs in medical journals. Such advertisements may increase prescriptions of new drugs at the expense of older treatments even when they have no added benefits, are more harmful, and are more expensive. The publication of medical advertisements therefore raises ethical questions related to editorial integrity.

**Methods:**

We conducted a descriptive cross-sectional study of all medical advertisements published in the *Journal of the Danish Medical Association* in 2015. Drugs advertised 6 times or more were compared with older comparators: (1) comparative evidence of added benefit; (2) Defined Daily Dose cost; (3) regulatory safety announcements; and (4) completed and ongoing post-marketing studies 3 years after advertising.

**Results:**

We found 158 medical advertisements for 35 prescription drugs published in 24 issues during 2015, with a median of 7 advertisements per issue (range 0 to 11). Four drug groups and 5 single drugs were advertised 6 times or more, for a total of 10 indications, and we made 14 comparisons with older treatments. We found: (1) ‘no added benefit’ in 4 (29%) of 14 comparisons, ‘uncertain benefits’ in 7 (50%), and ‘no evidence’ in 3 (21%) comparisons. In no comparison did we find evidence of ‘substantial added benefit’ for the new drug; (2) advertised drugs were 2 to 196 times (median 6) more expensive per Defined Daily Dose; (3) 11 safety announcements for five advertised drugs were issued compared to one announcement for one comparator drug; (4) 20 post-marketing studies (7 completed, 13 ongoing) were requested for the advertised drugs versus 10 studies (4 completed, 6 ongoing) for the comparator drugs, and 7 studies (2 completed, 5 ongoing) assessed both an advertised and a comparator drug at 3 year follow-up.

**Conclusions and relevance:**

In this cross-sectional study of medical advertisements published in the *Journal of the Danish Medical Association* during 2015, the most advertised drugs did not have documented substantial added benefits over older treatments, whereas they were substantially more expensive. From January 2021, the *Journal of the Danish Medical Association* no longer publishes medical advertisements.

**Supplementary Information:**

The online version contains supplementary material available at 10.1186/s41073-021-00111-9.

## Introduction

The pharmaceutical industry promotes prescription drugs in many ways, e.g. through sales visits to medical personnel, by arranging conferences with key opinion leaders, distributing reprints of studies published in prestigious medical journals, and through print advertisements [1]. Medical advertisements directed towards patients, often called” direct to consumer” advertisements, are allowed only in a few countries, including the United States and New Zealand. Opponents argue that these advertisements medicalise normal experiences and lead to unnecessary drug use [2]. Advocates argue that such advertisements increase patients’ autonomy and raise awareness about new treatments and diseases that would otherwise remain underdiagnosed and undertreated [3].

To our knowledge, medical advertisements for health care professionals, i.e. marketing content about new prescription drugs sponsored by pharmaceutical companies, are allowed in most countries and published in most medical journals. Researchers have recommended medical journals to abandon such advertisements arguing that they make medical doctors prescribe new drugs at the expense of older, cheaper, and often equivalent or better alternatives [4]. *PLOS Medicine* is likely one of the few medical journals that has chosen to not bring medical advertisements. At the journal’s inauguration, the editors stated they did not want to be part of a “cycle of dependency” with the pharmaceutical industry [5]. The International Committee of Medical Journal Editors (ICMJE) [6] and the World Association of Medical Editors (WAME) [7] recommend against medical advertisements to be juxtaposed to related scientific content. A case-control study of four international and three Russian medical journals (total of 214 issues) found 90 instances of advertisements published in an issue with closely related scientific content [8].

Medical advertisements may refer to data that do not substantiate claims or to data that is inaccessible [9–14], and they may present relative rather than absolute treatment effects, which could lead to exaggerated perceptions of treatment effects [15]. One third of antidepressant advertisements published in the *Journal of the Swedish Medical Association* between 1994 to 2003 violated the industry’s own code of conduct [16], and 82% of medical advertisements published in a sample of American medical journals in 2008 did not comply fully with the US Food and Drug Administration (FDA) advertising guideline [17]. A systematic review of the quality of medical advertisements reported that only 8% of the advertisements’ statements cited systematic reviews and 30% randomised trials [18]. Another systematic review found that exposure to pharmaceutical promotion was associated with increased prescription rates and costs, and lower quality of prescriptions, whereas there was no evidence of improved prescription quality, defined, for example, as adherence to prescription guidelines and appropriateness of prescriptions [19].

In this study we wanted to investigate which drugs were advertised in the *Journal of the Danish Medical Association* [20]*.* Additionally, for the most commonly advertised drugs, we wanted to assess the evidence for added benefit, cost, safety announcements, and drug regulator required post-marketing studies comparing with older prescription drugs for the same indication.

## Methods

We followed the Strengthening the Reporting of Observational Studies in Epidemiology (STROBE) guideline [21] for reporting our results.

### The medical journal

*The Journal of the Danish Medical Association* [20] is a general medical journal and a member of the International Committee of Medical Journal Editors (ICMJE). The journal is published biweekly in Danish and circulated in print to all members of the Danish Medical Association, which has approximately 30,000 medical doctors as members.

### Sample of advertisements

Two observers (KB and ALS) independently assessed all issues of the *Journal of the Danish Medical Association* published in 2015 (excluding special issues). A third author (KJ) arbitrated in case of disagreements. We extracted information from all medical advertisements published during 2015. We did not include advertisements for over-the-counter drugs, dietary supplements, or medical devices. We extracted the following information: trade name, generic name, drug class, indication, and sponsor. We categorised each advertisement according to the most relevant medical specialty. We looked up each advertisement’s indication (e.g. hypercholesterolaemia) in a Danish medical reference [22] and adopted its specialty categorisation (e.g. endocrinology).

In the European Union, medical advertisements must be juxtaposed by the product information, the Summary of Product Characteristics (SmPC) [23]. Some SmPCs may resemble actual advertisements by including logos, illustrations, tables, or descriptive text in addition to the mandated text. We therefore included those SmPCs that contained more than the legally required [23] information as advertisements. The results were summarised in Excel and are presented as summary statistics, i.e. percentages and medians.

### Advertisements coinciding with scientific content

We assessed whether advertisements for speciality drugs, i.e. drugs that are used and prescribed by specialists, appeared in issues with related scientific articles, e.g. narrative reviews related to the specialist drug.

### Comparison of most commonly advertised drugs versus older comparators

One author (KB) assessed the most advertised drugs with 6 or more advertisements. We grouped advertised drugs if they belonged to the same drug class, e.g. combination beta_2_-agonist and steroid formulations, or if they were advertised for the same specific indication, e.g. treatment of atrial fibrillation. We compared these most commonly advertised drugs or drug groups, with clinically relevant comparators. We defined the relevant comparators as single components of combination formulations, regular pill formulations of modified release formulations, or first-choice treatments for the advertised condition. See also the Supplementary file, eMethods. We made four analyses for these advertised drugs and their relevant comparators:

1. *Evidence for added benefits*

We searched for direct comparative evidence in Cochrane reviews, the Institute for Quality and Efficiency in Healthcare’s (IQWiG) assessment reports, FDA Medical Office Reviews and the European Medicines Agency’s (EMA) Public Assessment Reports, in that order. We categorised the evidence for added benefit of the advertised drug (for the advertised indication) relative to the comparator as ‘substantial added benefits’, ‘uncertain benefits’, ‘no added benefits’, or ‘no evidence’. See also Supplementary file, eMethods for details.

2. *Defined Daily Dose cost analysis*

We compared the advertised drugs’ Defined Daily Dose to those of the relevant comparators. We obtained prices from the Danish Medicines Agency [24].

3. *EMA and FDA safety announcements*

We searched the FDA Drug Safety Communication [25] archive and EMA’s medicines database [26] for safety announcements pertaining to the advertised drugs and their relevant comparators. We searched for announcements published within a three-year follow-up period after advertising between 2015 and 2018.

4. *Post-marketing studies*

We searched for drug regulator required post-marketing studies registered in the FDA Postmarket Requirements and Commitments database [27] and the European Union electronic Register of Post-Authorisation Studies [28]. We categorised the studies according to their status at 3 years of follow-up (December 2018) from advertising as: ‘completed’ (results reported before December 2018), or ‘ongoing’ (results reported, or planned to be reported, after December 2018). See the Supplementary file, eMethods for details.

## Results

### Summary results

During 2015, there were 158 medical advertisements for 35 different prescription drugs, published in 24 issues with a median of 7 per issue (range 0 to 11). Of the 158 advertisements, 35 (22%) were Summary of Product Characteristics that contained more than the legally required text. In two issues (no. 19 and no. 23), seven of the first nine pages were medical advertisements or SmPCs. In the two issues published during Danish summer holiday (July), there were no advertisements. See the full list of advertisements in Supplementary file, eTable 1. Drugs often prescribed in general practice were the most frequently advertised. Advertisements for pulmonology appeared most frequently (*n* = 57, 36%), followed by psychiatry (*n* = 32, 20%), analgesics (*n* = 17, 11%), endocrinology (*n* = 15, 8%), and urology (*n* = 8, 5%), see Supplementary file, eTable 2.

### Advertisements coinciding with scientific content

We found seven cases of advertisements for six specialist drugs appearing in issues with related scientific content, Supplementary file eTable 3. In three cases, the advertised drugs were directly mentioned in narrative reviews appearing in the same issue. In four cases, the advertisements appeared in issues with closely related scientific content, but the drugs were not directly mentioned.

### Comparison of most commonly advertised drugs versus older comparators

Four drug groups, combined beta_2_-agonist + steroid inhalations (three drugs), combined beta_2_-agonist + anticholinergic agent inhalations (three drugs), ADHD medications (two drugs), and new oral anticoagulants (two drugs) and five single drugs, modified-released paracetamol, vortioxetine, aripiprazole intramuscular depot, pneumococcal vaccine, and canagliflozin, were advertised ≥6 times during 2015, Table 1. The sample accounted for 118 (75%) of the 158 advertisements. We compared these frequently advertised drugs with older comparators in four analyses:

1. *Evidence for added benefits*

**Table 1:** Basic information on the most advertised drugs and drug groups

Advertised drug (n advertisements)	Brand name	EMA authorisation^**a**^	Marketing holder^**b**^
*Drug groups*			
**Beta2-agonist + steroid inhalations (28)**			
Vilanterol + fluticasone furoate (11)	Relvar Ellipta	Nov 2013	GlaxoSmithKline
Formoterol + budesonide (9)	DuoResp Spiromax	April 2014	Teva Pharma
Formoterol + fluticasone propionate (8)	Flutiform	June 2012	Norpharma
**Beta2-agonist + anticholinergic agents (26)**			
Olodaterol + tiotropium (18)	Spiolto Respimat	N/A^c^	Boehringer Ingelheim
Vilanterol + umeclidinium (5)	Anoro Ellipta	May 2014	GlaxoSmithKline
Formoterol + aclidinium bromide (3)	Duaklir Genuair	Nov 2014	Astra Zeneca
**ADHD medications (16)**			
Lisdexamfetamine (12)	Aduvanz	N/A^c^	Shire Pharmaceuticals
Atomoxetine (4)	Strattera	N/A^c^	Eli Lilly
**New oral anticoagulants (6)**			
Rivaroxaban (4)	Xarelto	June 2013	Bayer
Dabigatran (2)	Pradaxa	Aug 2011	Boehringer Ingelheim
*Single drugs*			
Paracetamol modified-release (15)	Panodil 665	N/A^c^	GlaxoSmithKline
Vortioxetine (8)	Brintellix	Dec 2013	Lundbeck
Aripiprazole intramuscular depot (7)	Abilify Maintena	Nov 2013	Otsuka/Lundbeck
Pneumococcal vaccine (6)	Prevenar 13	Jan 2010	Pfizer
Canagliflozin (6)	Invokana	Nov 2013	Janssen-Cilag

^a^We used the EMA year of approval as a proxy for regulatory approval in Denmark. ^b^The marketing holder is the pharmaceutical company that also sponsors the advertisements. ^c^We where not able to find relevant information on the EMA website. These drugs were likely approved through decentralised procedures, which means that a European national drug regulator authorised the drug and not EMA

The most advertised drugs (four drug groups, five single drugs) were advertised for 10 different indications and we made 14 comparisons with older comparators, Table 2. We used Cochrane systematic reviews (six comparisons), IQWiG reports (two), FDA reports (two), EMA report (one), and a single trial for one comparison. For two comparisons we found no evidence source. We included additional evidence outside of our stipulated search strategy for four comparisons (beta_2_-agonist + steroid combination for asthma, atomoxetine for adult ADHD, vortioxetine for depression, and pneumococcal vaccines for invasive pneumonia), Table 2.

**Table 2:** Comparative evidence for the most advertised drugs and drug groups

Advertised drug	Indication	Comparison	Reviewed evidence	Evidence categorisation
*Drug groups*				
**1. Beta2-agonist + steroid inhalations**	1. Asthma	1. Steroid inhalation only	Systematic review [29] + FDA analysis [30]^a^	Uncertain benefits
**-**	2. COPD	2. Beta_2_-agnoist inhalation only	Systematic review [31]	Uncertain benefits
**2. Beta2-agonist + anticholinergic agents**	2. COPD	3. Beta_2_-agonist only	Systematic review [32]	Uncertain benefits
-	2. COPD	4. Anticholinergic agent only	Systematic review [32]	Uncertain benefits
**3. ADHD medications**				
Lisdexamfetamine	3. ADHD	5. Methylphenidate	No evidence source	No evidence
Atomoxetine	3. ADHD	6. Methylphenidate	One clinical trial [33]^a^	No added benefits
**4. New oral anticoagulations**				
Rivaroxaban	4. Atrial fibrillation	7. Warfarin	FDA report [34]	No added benefits
Dabigatran	4. Atrial fibrillation	8. Warfarin	FDA report [35]	No added benefits
*Single drugs*				
5. Paracetamol modified-release	5. Pain	9. Regular paracetamol	No evidence source	No evidence
6. Vortioxetine	6. Depression	10. Duloxetine	Systematic review [36]^a^	No added benefits
7. Aripiprazole intramuscular depot	7. Schizophrenia	11. Aripiprazole oral tablet	EMA report [37]	Uncertain benefits
8. Pneumococcal vaccine	8. Pneumococcal pneumonia	12. Placebo	Systematic review [38] + clinical trial [39] ^a^	Uncertain benefits
9. Canagliflozin	9. Diabetes mellitus type 2 (single therapy)	13. Glimeride	IQWiG report [40]	No evidence
-	10. Diabetes mellitus type 2 (add-on to metformin)	14. Glimerpiride add-on to metformin	IQWiG report [40]	Uncertain evidence

*ADHD* attention deficit hyperactivity disorder; *COPD* chronic obstructive pulmonary disorder. ^a^For four comparisons we included evidence identified outside our stipulated search strategy. We have detailed this in the Supplement, eTable 8

The advertised drugs had evidence of ‘substantial added benefits’ compared to older relevant comparators in none (0%) of the comparisons, there were ‘uncertain benefits’ in seven (50%) comparisons, and evidence of ‘no added benefits’ of the advertised drugs in four (29%) comparisons. For three (21%) comparisons there was ‘no evidence’, Table 2. See the Supplementary file, ‘Evidence for the advertised drugs’ and eTable 4, for details on each comparison.

2. *Defined Daily Dose cost analysis*

The advertised drugs were two to 196 times (median of 6) more expensive measured as the Defined Daily Dose than the older comparators, Table 3. For unknown reasons, the Danish Medicines Agency did not report the Defined Daily Dose for inhalation combination drugs, and we could not make a price comparison for the pneumococcal vaccine against placebo.

3. *EMA and FDA announcements*

**Table 3:** Defined Daily Dose cost analysis

Advertised drug (defined daily dose)	Cost per Defined Daily Dose (DKK)	Older comparator	Cost per Defined Daily Dose (DKK)	Price ratio
Aripiprazole (400 mg as intramuscular injection)	102,41	Aripiprazole (15 mg as pill formulation)	0,52	196
Canagliflozin (300 mg)	10,41	Glimepiride (2 mg)	0,13	80
Rivaroxaban (20 mg)	21,66	Warfarin (7,5 mg)	3,49	6
Dabigatran (300 mg)	22,22	Warfarin (7,5 mg)	3,49	6
Atomoxetine (80 mg)	32,90	Extended-release methylphenidate (30 mg)^a^	6,71	5
Vortioxetine (10 mg)	7,65	Duloxetine (60 mg)	1,95	4
Lisdexamfetamine (30 mg)	17,23	Extended-release methylphenidate (30 mg)^a^	6,71	3
Paracetamol 665 mg (3990 mg)^b^	3,83	Paracetamol 500 mg (4000 mg)	1,75	2

*DKK* Danish Crowns (7,45 DKK approx. 1 Euro). Prices were obtained from the Danish Medicines Agency in March 2019.^a^Doses are reported as defined daily doses, and may not correspond to the used doses in a clinical setting. ^b^These are the 2017-prices, before the modified-release paracetamol was withdrawn from the EU market

Between 2015 and 2018, EMA and FDA made 11 announcements related to five of the advertised drugs, and one announcement also pertained to a relevant comparator (FDA’s warning on aripiprazole), Table 4. The FDA issued nine Drug Safety Communications (canagliflozin = 7; aripiprazole = 1; combined beta_2_-agonist and steroid inhalation formulation = 1) and EMA issued two Referrals (inhaled corticosteroids for chronic obstructive pulmonary disorder (COPD) and modified-release paracetamol). Most warnings related to new harms, whereas one safety announcement informed that combination beta_2_-agonist + steroid formulations did not increase the risk of serious asthma related outcomes. EMA’s Referral on modified-release paracetamol announced the drug’s withdrawal from the European market due to difficulties in managing drug overdoses.

4. *Post-marketing studies*

**Table 4:** EMA and FDA safety announcements published after advertising (2015–2018)

Drug	Clinical findings	Regulator	Regulatory action	Announcement
Canagliflozin	Increased risk of ketoacidosis	FDA	Undertaking further investigations (all SGLT2-inhibitors)	May 2015 [41]
Canagliflozin	Increased risk of bone fractures and decreased bone mineral density	FDA	Warning added to the FDA prescriber information	Sep 2015 [42]
Canagliflozin	Increased risk of ketoacidosis, urosepsis, and pyelonephritis	FDA^a^	Warning added to the FDA prescriber information (all SGLT2-inhibitors)	Dec 2015 [43]
Aripiprazole	Impulse-control problems (gambling, binge eat, shop, sex)	FDA	Warning added to the FDA prescriber information^a^	May 2016 [44]
Canagliflozin	Interim results: Increased risk of leg and foot amputations	FDA	Undertaking further investigations	May 2016 [45]
Canagliflozin	Risk of acute kidney injury	FDA	Revised warning on the FDA prescriber information	June 2016 [46]
Inhaled corticosteroids for COPD	Increased risk of pneumonia	EMA	Updated product information	July 2016 [47]
Canagliflozin	Increased risk of foot and leg amputation	FDA^a^	Addition of FDA boxed warning	May 2017 [48]
Combined beta_2_-agonist + steroid inhalation	No increased risk of serious asthma-related outcomes	FDA	Removal of FDA boxed warning	Dec 2017 [49]
Modified-release paracetamol	Difficulties in managing overdoses	EMA	Withdrawal of product from EU market	Dec 2017 [50]
Canagliflozin	Increased risk of necrotising fasciitis of the perineum	FDA	Warning added to the FDA prescriber information (all SGLT2-inhibitors)	Aug 2018 [51]

Announcements listed in chronological order. ^a^The warning pertained to all formulations of aripiprazole, both oral tablet and intramuscular injections. ^b^Similar referral issued by EMA

We identified 37 drug regulator requested post-marketing studies, 33 in the EU database and four in the FDA database. By December 2018, 12 studies were completed and 25 were ongoing, Table 5. Twenty (54%) post-marketing studies (7 completed, 13 ongoing) related to the advertised drugs (beta_2_-agonist + anticholinergic combinations = 4; canagliflozin = 5; combined beta_2_-agonist + steroid = 3; rivaroxaban = 3; vortioxetine = 2; aripiprazole, dabigatran, and lisdexamfetamin one each), Supplementary file eTable 5. Ten (27%) studies (4 completed, 6 ongoing) related to four comparator drugs (duloxetine = 5; methylphenidate = 2; umeclidinium = 2, metformin = 1), Supplementary file eTable 6. Finally, Seven (19%) studies (2 completed, 5 ongoing) assessed an advertised and a comparator drug (beta_2_-agonist and anticholinergic combinations versus single components = 4; dabigatran, rivaroxaban and warfarin = 2; paracetamol all formulations = 1), Supplementary file eTable 7.

**Table 5:** Post-marketing studies ongoing at three year follow-up after advertising (Dec 2018)

Drug	Study ID	Requested	Clinical question
***Advertised drugs***			
Aripiprazole IM	EUPAS21056	EMA	Specific harms (extrapyramidal symptoms)
Canagliflozin	EUPAS27670	EMA	Specific harm (lower limb amputations)
Canagliflozin	NDA 204042 commitment no. 1	FDA	Specific harm (ketoacidosis)
Canagliflozin	NDA 204042 commitment no. 3	FDA	Specific harm (various conditions)
Fluticasone propionate / formoterol	EUPAS3702^a^	MHRA	Benefits and harms
Lisdexamfetamin	EUPAS20546	EMA	Specific harm (cardiovascular events)
Rivaroxaban	EUPAS11299, EUPAS9895, EUPAS11141, and EUPAS11145	EMA	Specific harms (bleeding events and liver disease)
Tiotropium / olodaterol	EUPAS14273	Japan	Long-term benefits and harms
Tiotropium / olodaterol	EUPAS21574	EMA	Specific harms (cardiovascular)
Tiotropium / olodaterol	EUPAS14956	South Korea	Benefits and harms
Umeclidinium/ vilanterol	EUPAS9868	Japan	Benefits and harms
Umeclidinium/ vilanterol	EUPAS11397	South Korea	Benefits and harms
Vortioxetine	NDA 204447 commitment no. 6	FDA	Benefits and harms
Vortioxetine	EUPAS19199	EMA	Clinical use and several specific harms
***Comparator drugs***			
Duloxetine	EUPAS20253^b^	United States	Specific harms (maternal and fetal harms)
Duloxetine	NDA 21427 commitment no. 2	FDA	Specific harms (maternal and fetal harms)
Methylphenidate	EUPAS4551^c^	EMA	Harms
Methylphenidate	EUPAS3985^c^	EMA	Long-term harms
Umeclidinium	EUPAS14947	South Korea	Benefits and harms
Umeclidinium	EUPAS10224	Japan	Benefits and hams
***Advertised drugs and comparator drugs***			
Aclidinium and aclidinium/ formoterol	EUPAS6559	EMA	Clinical use
Aclidinium and aclidinium/ formoterol	EUPAS13616	EMA	Specific harms (cardiovascular and mortality)
Dabigatran and rivaroxaban, versus warfarin	EUPAS13017	France	Benefits and harms
Olodaterol and olodaterol/tiotropium	EUPAS21574	EMA	Specific harms (cardiovascular events)
Umeclidinium and umeclidinium/ vilanterol	EUPAS10316	EMA	Specific harms (cardiovascular and cerebrovascular events)

*EMA* European Medicines Agency; *FDA* US Food and Drug Administration; *IM* Intramuscular. ^a^The study was scheduled to report data in 2015, but data had not been submitted. ^b^This study may likely be the same. ^c^The two methylphenidate studies were planned to finish in 2014 and 2015, but they were listed as ongoing since data had not been reported on the website

The postmarketing studies assessed specific harms (24, 65%), benefits and harms (11, 22%), and prescription patterns (2, 5%), e.g. off-label use. All studies had an observational design, e.g. pharmacovigilance and cohort studies, except one randomised clinical trial for the antidepressant vortioxetine.

## Discussion

### Key results

To our knowledge, this is the first cross-sectional study to assess all medical advertisements published in a general medical journal throughout a calendar year. We judged that none of the most frequently advertised drugs were supported by evidence of ‘substantial added benefits’ compared to relevant comparators. This corresponds with recent reports that the evidence for the majority of new cancer drugs [52] and newly authorised drugs in Germany demonstrate little or no added patient-relevant benefits over existing treatments [53]. Our analyses highlight, perhaps unsurprisingly, that advertised drugs were substantially more expensive than existing drugs on the market, which may be important in the light of the poor evidence for added value to the patients. Finally, we found that there were numerically more safety announcements issued regarding newly identified harms and more uncompleted post-marketing studies addressing potential harms related to the advertised drugs at three years of follow-up. This may indicate a larger uncertainty related to the clinical use of newer drugs compared to older comparators. In general, our study seems to add to the existing literature that medical advertisements directed towards healthcare professionals may not have beneficial effects but may have important negative effects.

### Interpretation

We published an abridged version of this paper as an opinion piece in the *Journal of the Danish Medical Association* in 2018 in Danish [54] and encouraged the Danish Medical Association to ban medical advertisements. On 19 June 2020, the Delegation of the Danish Medical Association voted to abolish medical advertisements [55] and, to our knowledge, this may be the first national medical association to make such a decision. We do not know if, and if so, to what degree, our work [54, 56] and advocacy for banning medical advertisements influenced this decision.

Nevertheless, an important question remains whether other medical associations, medical societies, and their respective medical journals could and should follow suit and abolish such advertisements. We are not aware of discussions regarding such a ban of advertisements for healthcare professionals in Europe. In contrast, on several occasions the introduction of ‘direct to consumer’ advertisements have been proposed to the European Commission [57, 58]. Similarly, we are not aware of discussions in the United States related to a ban of these advertisements directed at healthcare professionals, despite pharmaceutical companies spending more money on marketing directed at healthcare professionals than on ‘direct to consumer’ marketing. In 2016, a total of $20.3 billion was spent on marketing directed at healthcare professionals in the US compared to $9.6 billion on “direct to consumer” marketing [59]. Interestingly, it has long been debated in the US whether ‘direct to consumer’ advertisements should be banned [60] and in 2015, the American Medical Association advocated for such a ban stating these advertisements increase the use of more expensive and less effective treatments [61]. The same case could likely be made for advertisements directed at healthcare professionals.

Revenue from medical advertisements is intrinsic to the current biomedical publishing model, along with other revenues, such as sales of re-prints [62]. A cross-sectional study of six medical societies in 1996 estimated that advertising revenue accounted for 2 to 31% of the associations’ total revenue [63]. Major medical publishers report very large profit margins, e.g. Informa (who owns Taylor & Francis) reported an operating profit of £933 million (32%) of a total revenue of £2,9 billion in 2019 (p. 161) [64]. Elsevier reported an adjusted operating profit of £982 million (37%) of a total revenue of £2,6 billion in 2019 [65]. Interestingly, only 2% of this revenue came from advertising whereas subscription fees was the major source of income (p. 16) [65]. An informed guess is therefore that abolishing of medical advertisements is economically feasible for major journals and publishers.

The impact on public health of advertisement to healthcare professionals may also not be trivial. The current ‘opioid crisis’ has been associated with marketing of OxyContin directed towards doctors falsely highlighting its low potential for addiction [66]. An American observational study [67] reported a positive association between opioid marketing directed at doctors, prescription rates, and overdose mortality, and registry studies [68, 69] have suggested that a major cause for the recent decrease in the US overall life expectancy is opioid-related mortality. The American Centers for Disease Control and Prevention estimates that nearly 500.000 Americans died because of an opioid related overdose between 1999 and 2019 [70].

### Limitations

Our study has several limitations, most importantly the lack of preregistration and specification of our methodology, including pre-defining how to select the comparator drugs. We only assessed advertisements during one year and in one medical journal, yielding a relatively small sample of issues and advertisements, which prevented us from making inferential statistics.

We grouped drugs if they belonged to the same drug class, and some comparisons might therefore have been affected. However, regardless of our preferred ‘unit of analysis’, drugs lumped into groups or individual assessments, our conclusions would likely be similar. For example, the most advertised drug, the combination drug tiotropium/olodaterol indicated for COPD, was included in the ‘beta_2_-agonist and anti-cholinergic combination’ group. According to an IQWiG report [71], this drug has “proof of lesser benefit” compared to a beta_2_-agonist or an anti-cholinergic agent alone. Individual drug assessments might even have led to more critical assessments.

While evidence categorisation always contains a degree of subjectivity, we believe that our methods and analyses are transparently reported and that other researchers would likely come to the same conclusions. Some might consider our inclusion of additional evidence outside our stipulated search strategy for some comparisons as unsystematic and potentially biased. It is important to note that the inclusion of this additional evidence did not change our evidence classifications. On the contrary, these efforts illustrate how difficult it may be to obtain the best and most complete available evidence. In fact, difficulties in identifying relevant direct comparisons might illustrate a general problem in the current regulatory drug approval system [53], rather than being a limitation to our project. We consider it an advantage that our evidence categorisation was based on systematic reviews and regulatory drug reports, which often include raw data from pivotal trials.

There might be other approaches to assessing the comparative safety of new versus older drugs than using safety announcements and post-marketing studies as the metric. The number of post-marketing studies does not necessarily indicate a greater uncertainty related to the use of new drugs compared to the older comparators, but rather that new drugs are subject to more scrutiny upon, and after, authorisation. Nevertheless, the higher number of unfinished post-marketing studies and safety announcements imply an uncertainty related to the prescription of these new drugs that did not apply to older comparators. Importantly, these uncertainties may not be conveyed to patients until many years after these drugs have been advertised heavily. Finally, we searched for post-marketing studies related to the single components of the combination inhalation formulations, but we did not search for all authorised single component beta_2_-agonists, anticholinergic agents, and steroid drugs. However, it is unlikely this would have impacted our overall results.

### Generalisability

We assessed a single medical journal during one calendar year and the results may therefore not be generalisable. However, the *Journal of the Danish Medical Association* is a national general medical journal circulated to all members of the Danish Medical Association across specialties and settings, which makes us believe that the assessed sample of advertised drugs was broad and may reflect well on content in other general medical journals. Our Defined Daily Dose cost analysis applies to Denmark and only at the time of analysis. Cost difference-ratios will be different in other countries and fluctuate over time [72].

We did not assess all advertised drugs, which were beyond the resources available for this research project. The assessed cohort of advertised drugs included commonly used drugs and we believe that our findings likely are transferable to other general medical journals.

## Conclusion

In this cross-sectional study of medical advertisements published in a Danish general medical journal during 2015, we did not find evidence of substantial added benefits of the most advertised drugs over older comparators. The advertised drugs were substantially more expensive and likely related to more uncertain use measured on the number of EMA and FDA safety announcements and unfinished post-marketing studies at three years follow-up after the advertisement. *The Journal of the Danish Medical Association* stopped publishing medical advertisements from 2021.

## Supplementary Information

**Additional file 1.**

## Data Availability

The full list of advertisements is available in in the supplement. The issues of *the Journal of the Danish Medical Association* are available upon subscription from http://ugeskriftet.dk.
