# Correction to: Cross-sectional study of medical advertisements in a national general medical journal: evidence, cost, and safe use of advertised versus comparative drugs

**DOI:** 10.1186/s41073-021-00114-6

**Published:** 2021-06-11

**Authors:** Kim Boesen, Anders Lykkemark Simonsen, Karsten Juhl Jørgensen, Peter C. Gøtzsche

**Affiliations:** 1 Nordic Cochrane Centre, Rigshospitalet Dept. 7811, 2100 Copenhagen, Denmark; 2 Current address: Meta Research Innovation Center Berlin (METR IC-B), Berlin Institute of Health, Charité Universitätsmedizin, QUEST Center for Transforming Biomedical Research, Berlin, Germany; 3 Centre for Evidence-Based Medicine (CEBMO) and Cochrane Denmark, Dept. Clinical Research, University of Southern Denmark, Odense, Denmark; 4 Open Patient data Exploratory Network (OPEN), Odense University Hospital, Odense, Denmark; 5 Institute for Scientific Freedom, 2970 Copenhagen, Denmark

**Correction to: Res Integr Peer Rev 6, 8 (2021)**

**https://doi.org/10.1186/s41073-021-00111-9**

Following publication of the original article [1], the authors noted that an incorrect file for Supplementary file 1 had been published. The corrected version of Supplementary file 1 is attached to this Correction and it has also been updated in the original article, accordingly.

## Supplementary Information

**Additional file 1.**
