# Supplementary material for: Cross-sectional study of medical advertisements in a national general medical journal: evidence, cost, and safe use of advertised versus comparative drugs
Source: Res Integr Peer Rev. 2021 May 10;6:8. doi: 10.1186/s41073-021-00111-9 (PMC8108346; doi:10.1186/s41073-021-00111-9)
Supplement: Supplementary file 1 — Additional file 1. [file 41073_2021_111_MOESM1_ESM.pdf]

Online Supplement

Boesen K, Simonsen AL, Jørgensen KJ, Gøtzsche PC. Cross-sectional study of medical advertisements in a general medical journal: Evidence, cost, and safe use of advertised versus comparative drugs. *Research Integrity and Peer Review* 2021;6:8.
https://doi.org/10.1186/s41073-021-00111-9.

Table of Contents

eMethods 2
Defining the clinically relevant comparator 2
Searching for comparative evidence 3
Categorising the evidence 3
Searching for safety announcements 5
Searching for post-marketing studies 5
eResults 7
eTable 1. Full list of medical advertisements. 7
eTable 2. Medical advertisements separated into specialty and generic drug 14
eTable 3. Coincidences of drug advertisements and related scientific content 16
Evidence for the advertised drugs 17
eTable 4. Conclusions or abstracts to support evidence categorisation 22
eTable 5. Post-marketing studies of advertised drugs 29
eTable 6. Post-marketing studies for comparator drugs 34
eTable 7. Post-marketing studies including both advertised and comparator drugs 37
eTable 8. Search overview for comparative evidence 40
Detailed search results 42
eTable 9. Comments on the search strategy 47
eReferences 49

eMethods

Defining the clinically relevant comparator

We compared the evidence of the most frequently advertised drugs or drug groups with older comparators. We defined the relevant comparators accordingly:

Combination formulations

We compared with the clinically relevant individual single-agent components: for the combination therapy of a long acting beta2-agonist and a steroid for asthma we chose steroid alone as the comparator. For chronic obstructive pulmonary disorder (COPD) we chose long acting beta2-agonists alone. For the combination therapy of long acting beta2-agonist and anticholinergic agents for COPD we chose both single agents as comparators.

Modified formulations

For intramuscular injection aripiprazole and modified-release paracetamol we compared with the regular oral tablet formulations.

Other comparisons

For dabigatran and rivaroxaban for use in atrial fibrillation we chose warfarin, which has been the preferred drug for decades. For canagliflozin for use in diabetes type 2 we chose metformin, since the drug is indicted as a substitute for metformin, and glimepiride since the drug is indicated as an add-on to metformin. For vortioxetine for use in depression, we chose duloxetine because this was the active comparator in the pivotal trials. Venlafaxine was also included as an active comparator in the pivotal trials, and one could also argue that a SSRI, such as citalopram, would have been a more appropriate comparator. For atomoxetine and lisdexamfetamin for attention deficit hyperactivity disorder (ADHD) in adults we chose methylphenidate in the extended-release formulation. For the pneumococcal vaccine for the prevention of invasive pneumococcal pneumonia we chose placebo.

Searching for comparative evidence

We established a hierarchy for identifying comparative evidence. We preferably used Cochrane reviews to assess the evidence of the advertised drugs against older available treatments. Since most advertised drugs were newly approved drugs or formulations, we could not identify Cochrane reviews for all comparisons. We therefore sought evidence from the German Institute for Quality and Efficiency in Healthcare (IQWiG) projects' database. IQWiG makes drug assessments of newly authorised drugs in Germany based on Clinical Study Reports, submitted for marketing authorisation. IQWiG categorises the added benefit of the new drugs compared to standard care. If we could not identify relevant IQWiG reports, we searched FDA and EMA databases (in that order) of Drug Approval Packages and Public Assessment Reports for relevant pivotal trials or other comparable evidence.

- Cochrane Library (https://www.cochranelibrary.com/).
- IQWiG projects' database (https://www.iqwig.de/en/projects-results/projects.1057.html).
- FDA's database of approved drugs (https://www.accessdata.fda.gov/scripts/cder/daf/).
- EMA's database of Public Assessment Reports (https://www.ema.europa.eu/en/medicines).

Categorising the evidence

We categorised the comparative evidence of advertised drugs versus older comparative drugs based on four criteria:

1) Was there evidence from randomised trials?
2) Were there important methodological limitations of the trials that hindered the interpretation of the results?
3) Was the advertised drug reported as being better than the older comparator?
4) Was the reported difference clinically relevant, such as quality of life, mortality, or hard functional outcomes, rather than surrogate outcomes.

Based on these 4 criteria we categorised the evidence as:

‘Added substantial benefits’

The advertised drug had been tested directly against an older available treatment, or placebo, in a randomised clinical trial and was assessed as demonstrating *clinically relevant* benefits, either in terms of better beneficial outcomes, fewer harms, or both.

‘Uncertain benefits’

The advertised drug was either 1) tested directly against an older available treatment, but the reported effects were small and/or the outcome was irrelevant for patients, or 2) there were methodological limitations of the relevant trials, which limited the interpretation of the results.

‘No added benefit’

The advertised drug had been tested directly against a relevant comparator and it was assessed as being similar to it, or inferior.

‘No evidence’

We were either unable to find evidence of comparisons with the relevant comparator in a randomised trial, or it was explicitly reported that the drugs had not been directly compared.

We took inspiration from the IQWiG method of categorising evidence of newly approved drugs (Wieseler et al. 2019, https://doi.org/10.1136/bmj.l4340). We modified the evidence categorisation to reflect that we used secondary assessments, i.e. systematic reviews and regulatory documents, rather than having access to the Clinical Study Reports ourselves. We therefore joined the categories used by IQWiG (‘minor’, ‘considerable’, and ‘major benefits’) into one category “added substantial benefits” since we would likely not be able to make such differentiated assessments.

Searching for safety announcements

We systematically searched safety warnings published in FDA's archive of Drug Safety Communications (https://www.fda.gov/Drugs/ucm199082.htm) and EMA's database for Referrals (https://www.ema.europa.eu/en/medicines) issued between 2015 and 2018 related to the most advertised drugs or drug groups and the relevant comparators. We included communications that informed about specific drugs and also communications regarding relevant drug classes, e.g. SGLT2-inhibitors or combined beta2-agonist and steroid inhalation formulations.

If FDA and EMA issued similar warnings, we included the earliest issued warning to avoid double counting. We included warnings about the safe use of these drugs only. We did not include warnings about practicalities, such as drug name changes or other practical matters.

Searching for post-marketing studies

We systematically searched FDA's Postmarket Requirement and Commitments (PMC) database (https://www.accessdata.fda.gov/scripts/cder/pmc/index.cfm) and the European Union electronic Register of Post-Authorisation Studies (EU PAS Register) (http://www.encepp.eu/encepp/studySearch.htm) for post-marketing studies requested by drug regulators for the most advertised drugs or drug groups and their relevant comparators. For FDA's PMC we searched for studies listed as CDER (Center for Drug Evaluation and Research) but not as CBER (Center for Biologics Evaluation and Research). The EU PAS Register is not limited to studies requested by EMA but includes studies from several national regulators.

We included all post-authorised randomised clinical trials and observational studies, regardless if they had already completed or were ongoing scheduled to complete, or have to a final report, after January 2019. Pharmacokinetic studies or other non-clinical studies were not considered. We did not include studies conducted in age groups or in populations, for which the drug was not already approved (such as studies required to be conducted with children under the FDA Pediatric Research Equity Act). We did not include post-marketing studies of single agents, if these agents were only advertised in combination formulations

(such as the combination inhalation agents), and the post-marketing study did not include, or compare with, the combination formulation. We also did not include studies that assessed the advertised or comparator drugs for other than the advertised indications.

We searched for the studies using the drugs' generic names.

We ran the first search in January 2019 and re-ran the search during peer review in Dec 2020. We defined the 31 December 2018 as the cut off date for completion to align this analysis with the three year follow-up cut-off for the safety warnings analysis. If the studies ended after this date, they were categorised as 'ongoing'.

eResults

eTable 1. Full list of medical advertisements.

All medical advertisements and Summary of Product Characteristics included in the analysis published in the *Journal of the Danish Medical Association* in 2015.

Issue	Page	Trade name	Generic name	Indication	Medical speciality	Advertiser	Comments
1	2	Spiriva Respimat / Striverdi Respimat	Tiotropium / olodaterol	COPD + asthma / COPD	Pulmonology	Boehringer Ingelheim	
1	12	Panodil 665 mg	Paracetamol	Pain		GlaxoSmithKline	
1	100	Symbicort Forte Turbohaler	Budesonide + formoterol	COPD + asthma	Pulmonology	AstraZeneca	
2	102	DuoResp Spiromax	Budesonide + formoterol	COPD + asthma	Pulmonology	TEVA Pharma	
3	202	Valdoxan	Agomelatine	Depression	Psychiatry	Servier	
3	223	Dymista	Azelastine + fluticasone	Allergic rhinitis	Otorhinolaryngolo gy	MEDA	
3	229	DuoResp Spiromax	Budesonide + formoterol	COPD + asthma	Pulmonology	TEVA Pharma	
3	234	Panodil 665 mg	Paracetamol	Pain		GlaxoSmithKline	
3	295	Flutiform	Fluticasone propionate + formoterol	Asthma	Pulmonology	Norpharma	Summary of product characteristics with logo, picture, and tables.
3	300	Flutiform	Fluticasone propionate + formoterol	Asthma	Pulmonology	Norpharma	
4	302	Abilify Maintena	Aripiprazole	Schizophrenia	Psychiatry	Otsuka + Lundbeck	
4	306	Duaklir Genuair	Aclidinium bromide + formoterol	COPD	Pulmonology	AstraZeneca	
4	317	Panodil 665 mg	Paracetamol	Pain		GlaxoSmithKline	
4	400	Brintellix	Vortioxetine	Depression	Psychiatry	Lundbeck	
5	402	Duaklir Genuair	Aclidinium bromide + formoterol	COPD	Pulmonology	AstraZeneca	
5	406	Acnatac	Clindamycin + tretinoin	Acne vulgaris	Dermatology	MEDA	
5	425	Panodil 665 mg	Paracetamol	Pain		GlaxoSmithKline	
5	499	DuoResp Spiromax	Budesonide + formoterol	COPD + asthma	Pulmonology	TEVA Pharma	
5	500	Brintellix	Vortioxetine	Depression	Psychiatry	Lundbeck	

6	502	Duaklir Genuair	Aclidinium bromide + formoterol	COPD	Pulmonology	AstraZeneca	
6	506	Selexid	Pivmecillinam	Urinary tract infection	Urology	LEO Pharma	
6	511	Panodil 665 mg	Paracetamol	Pain		GlaxoSmithKline	
6	512	Dymista	Azelastine + fluticasone	Allergic rhinitis	Otorhinolaryngolo gy	MEDA	
6	524	Atozet	Atorvastatin + ezetimibe	Hypercholesterolemia	Endocrinology	MSD	
6	532	Acnatac	Clindamycin + tretinoin	Acne vulgaris	Dermatology	MEDA	
6	597	Norspan	Buprenorphine	Pain		Norpharma	Summary of product characteristics with logo.
6	599	Norspan	Buprenorphine	Pain		Norpharma	
6	600	Abilify Maintena	Aripiprazole	Schizophrenia	Psychiatry	Otsuka + Lundbeck	
7	611	Flutiform	Fluticasone propionate + formoterol	Asthma	Pulmonology	Norpharma	
7	612	Flutiform	Fluticasone propionate + formoterol	Asthma	Pulmonology	Norpharma	Summary of product characteristics with logo, picture, and tables.
8	702	Brintellix	Vortioxetine	Depression	Psychiatry	Lundbeck	
8	706	Dymista	Azelastine + Fluticasone	Allergic rhinitis	Otorhinolaryngolo gy	MEDA	
8	711	Flutiform	Fluticasone propionate + formoterol	Asthma	Pulmonology	Norpharma	
8	712	Flutiform	Fluticasone propionate + formoterol	Asthma	Pulmonology	Norpharma	Summary of product characteristics with logo, picture, and tables.
8	719	Panodil 665 mg	Paracetamol	Pain		GlaxoSmithKline	
8	727	Betmiga	Mirabegron	Overactive bladder	Urology	Astellas Pharma	
8	729	Atozet	Atorvastatin + ezetimibe	Hypercholesterolemia	Endocrinology	MSD	
8	797	Xarelto	Rivaroxaban	Prevention of thromboembolic events	Cardiology	Bayer	Summary of product characteristics with logo and tables.
8	800	Xarelto	Rivaroxaban	Prevention of thromboembolic events	Cardiology	Bayer	
9	802	Abilify Maintena	Aripiprazole	Schizophrenia	Psychiatry	Otsuka + Lundbeck	
9	806	Betmiga	Mirabegron	Overactive bladder	Urology	Astellas Pharma	
9	812	Atozet	Atorvastatin + ezetimibe	Hypercholesterolemia	Endocrinology	MSD	
9	897	Xarelto	Rivaroxaban	Prevention of thromboembolic events	Cardiology	Bayer	Summary of product characteristics with logo and tables.
9	900	Xarelto	Rivaroxaban	Prevention of thromboembolic events	Cardiology	Bayer	
10	902	Brintellix	Vortioxetine	Depression	Psychiatry	Lundbeck	

10	906	Dymista	Azelastine + fluticasone	Allergic rhinitis	Otorhinolaryngolo gy	MEDA	
10	911	DuoResp Spiromax	Budesonide + formoterol	COPD + asthma	Pulmonology	TEVA Pharma	
10	919	Panodil 665 mg	Paracetamol	Pain		GlaxoSmithKline	
10	936	Selexid	Pivmecillinam	Urinary tract infection	Urology	LEO Pharma	
10	1000	Abilify Maintena	Aripiprazole	Schizophrenia	Psychiatry	Otsuka + Lundbeck	
11	1002	Esbriet	Pirfenidone	Idiopathic pulmonary fibrosis	Pulmonology	Roche	
11	1004	Esbriet	Pirfenidone	Idiopathic pulmonary fibrosis	Pulmonology	Roche	Summary of product characteristics with logo.
11	1004	Atozet	Atorvastatin + ezetimibe	Hypercholesterolemia	Endocrinology	MSD	
11	1006	DuoResp Spiromax	Budesonide + formoterol	COPD + asthma	Pulmonology	TEVA Pharma	
11	1011	Brintellix	Vortioxetine	Depression	Psychiatry	Lundbeck	
11	1019	Zydelig	Idelalisib	Chronic lymphatic leukaemia and follicular lymphoma	Haematology	Gilead	
11	1029	Abilify Maintena	Aripiprazole	Schizophrenia	Psychiatry	Otsuka + Lundbeck	
11	1036	Betmiga	Mirabegron	Overactive bladder	Urology	Astellas Pharma	
11	1097	Invokana	Canagliflozin	Diabetes mellitus type 2 (mono therapy and as add-on)	Endocrinology	Janssen-Cilag	Summary of product characteristics with logo.
11	1100	Invokana	Canagliflozin	Diabetes mellitus type 2 (mono therapy and as add-on)	Endocrinology	Janssen-Cilag	
12	1102	Simponi	Golimumab	Rheumatoid arthritis, psoriatic arthritis, ankylosing spondylitis, and ulcerative colitis	Rheumatology	MSD	
12	1106	Flutiform	Fluticasone propionate + formoterol	Asthma	Pulmonology	Norpharma	Summary of product characteristics with logo, picture, and tables.
12	1107	Flutiform	Fluticasone propionate + formoterol	Asthma	Pulmonology	Norpharma	
12	1108	Dymista	Azelastine + fluticasone	Allergic rhinitis	Otorhino- laryngology	MEDA	
12	1113	Panodil 665 mg	Paracetamol	Pain		GlaxoSmithKline	
12	1122	Harvoni	Ledipasvir + sofosbuvir	Hepatitis C	Gastroenterology	Gilead	
12	1123	Harvoni	Ledipasvir + sofosbuvir	Hepatitis C	Gastroenterology	Gilead	Summary of product characteristics with logo.
12	1205	Invokana	Canagliflozin	Diabetes mellitus type 2 (mono therapy and as add-on)	Endocrinology	Janssen-Cilag	Summary of product characteristics with logo.
12	1208	Invokana	Canagliflozin	Diabetes mellitus type 2 (mono therapy and as add-on)	Endocrinology	Janssen-Cilag	

13	1210	Toujeo	Insulin glargine	Diabetes mellitus (adults)	Endocrinology	Sanofi-Aventis	
13	1305	Strattera	Atomoxetine	ADHD (children and adults)	Psychiatry	Eli Lilly	Summary of product characteristics with logo.
13	1308	Strattera	Atomoxetine	ADHD (children and adults)	Psychiatry	Eli Lilly	
14	No medical advertisements						
15	No medical advertisements						
16	1478	Simponi	Golimumab	Rheumatoid arthritis, psoriatic arthritis, ankylosing spondylitis, and ulcerative colitis	Rheumatology	MSD	
17	1562	Toujeo	Insulin glargine	Diabetes mellitus (adults)	Endocrinology	Sanofi-Aventis	
17	Loose leaf	Spiolto respimat	Tiotropium + olodaterol	COPD	Pulmonology	Boehringer Ingelheim	
17	Loose leaf	Spiolto respimat	Tiotropium + olodaterol	COPD	Pulmonology	Boehringer Ingelheim	Summary of product characteristics with logo and picture.
17	1566	Spiolto respimat	Tiotropium + olodaterol	COPD	Pulmonology	Boehringer Ingelheim	
17	1660	Brintellix	Vortioxetine	Depression	Psychiatry	Lundbeck	
18	1662	Toujeo	Insulin glargine	Diabetes mellitus (adults)	Endocrinology	Sanofi-Aventis	
18	Loose leaf	Spiolto respimat	Tiotropium + olodaterol	COPD	Pulmonology	Boehringer Ingelheim	
18	Loose leaf	Spiolto respimat	Tiotropium + olodaterol	COPD	Pulmonology	Boehringer Ingelheim	Summary of product characteristics with logo and picture.
18	1666	Aduvanz	Lisdexamfetamine	ADHD (adults)	Psychiatry	Shire	
18	1670	Aduvanz	Lisdexamfetamine	ADHD (adults)	Psychiatry	Shire	Summary of product characteristics with logo.
18	1673	Panodil 665 mg	Paracetamol	Pain		GlaxoSmithKline	
18	1690	Ubiquinon		Treatment of the heart		Pharma Nord	Advertisement for seminar.
19	1762	Strattera	Atomoxetine	ADHD (children and adults)	Psychiatry	Eli Lilly	
19	Loose leaf	Spiolto respimat	Tiotropium + olodaterol	COPD	Pulmonology	Boehringer Ingelheim	
19	Loose leaf	Spiolto respimat	Tiotropium + olodaterol	COPD	Pulmonology	Boehringer Ingelheim	Summary of product characteristics with logo and picture.
19	1764	Spiolto respimat	Tiotropium + olodaterol	COPD	Pulmonology	Boehringer Ingelheim	Summary of product characteristics with logo.
19	1766	Aduvanz	Lisdexamfetamine	ADHD (adults)	Psychiatry	Shire	Summary of product characteristics with logo.
19	1767	Aduvanz	Lisdexamfetamine	ADHD (adults)	Psychiatry	Shire	
19	1768	Spiolto respimat	Tiotropium + olodaterol	COPD	Pulmonology	Boehringer Ingelheim	
19	1779	Panodil 665 mg	Paracetamol	Pain		GlaxoSmithKline	
19	1794	Selexid	Pivmecillinam	Urinary tract infection	Urology	LEO Pharma	

19	1860	Brintellix	Vortioxetine	Depression	Psychiatry	Lundbeck	
20	1862	Toujeo	Insulin glargine	Diabetes mellitus (adults)	Endocrinology	Sanofi-Aventis	
20	Loose leaf	Spiolto respimat	Tiotropium + olodaterol	COPD	Pulmonology	Boehringer Ingelheim	
20	Loose leaf	Spiolto respimat	Tiotropium + olodaterol	COPD	Pulmonology	Boehringer Ingelheim	Summary of product characteristics with logo and picture.
20	1866	Aduvanz	Lisdexamfetamine	ADHD (adults)	Psychiatry	Shire	Summary of product characteristics with logo.
20	1867	Aduvanz	Lisdexamfetamine	ADHD (adults)	Psychiatry	Shire	
20	1868	Simponi	Golimumab	Rheumatoid arthritis, psoriatic arthritis, ankylosing spondylitis, and ulcerative colitis	Rheumatology	MSD	
20	1881	Panodil 665 mg	Paracetamol	Pain		GlaxoSmithKline	
20	1960	Prevenar 13	Pneumococcal vaccine	Prevention of pneumonia	Infectious disease	Pfizer	
21	1962	Relvar Ellipta	Fluticasone furoate + vilanterol	COPD + asthma	Pulmonology	GSK + Theravance	
21	Loose leaf	Spiolto respimat	Tiotropium + olodaterol	COPD	Pulmonology	Boehringer Ingelheim	
21	Loose leaf	Spiolto respimat	Tiotropium + olodaterol	COPD	Pulmonology	Boehringer Ingelheim	Summary of product characteristics with logo and picture.
21	1964	Relvar Ellipta	Fluticasone furoate + vilanterol	COPD + asthma	Pulmonology	GlaxoSmithKline + Theravance	Summary of product characteristics with logo, picture, and table.
21	1966	Prevenar 13	Pneumococcal vaccine	Prevention of pneumonia	Infectious disease	Pfizer	
21	2057	Invokana	Canagliflozin	Diabetes mellitus type 2 (mono therapy and as add-on)	Endocrinology	Janssen-Cilag	Summary of product characteristics with logo.
21	2060	Invokana	Canagliflozin	Diabetes mellitus type 2 (mono therapy and as add-on)	Endocrinology	Janssen-Cilag	
22	2062	Relvar Ellipta	Fluticasone furoate + vilanterol	COPD + asthma	Pulmonology	GlaxoSmithKline + Theravance	
22	2064	Relvar Ellipta	Fluticasone furoate + vilanterol	COPD + asthma	Pulmonology	GlaxoSmithKline + Theravance	Summary of product characteristics with logo, picture, and table.
22	2066	Simponi	Golimumab	Rheumatoid arthritis, psoriatic arthritis, ankylosing spondylitis, and ulcerative colitis	Rheumatology	MSD	
22	2071	Fucithalmic	Fusidic acid	Eye infection	Ophthalmology	AMCo	
22	2073	Prevenar 13	Pneumococcal vaccine	Prevention of pneumonia	Infectious disease	Pfizer	
22	2086	Abilify Maintena	Aripiprazole	Schizophrenia	Psychiatry	Otsuka + Lundbeck	
22	2091	Panodil 665 mg	Paracetamol	Pain		GlaxoSmithKline	
22	2093	Betmiga	Mirabegron	Overactive bladder	Urology	Astellas Pharma	

22	2110	Aduvanz	Lisdexamfetamine	ADHD (adults)	Psychiatry	Shire	Summary of product characteristics with logo.
22	2111	Aduvanz	Lisdexamfetamine	ADHD (adults)	Psychiatry	Shire	
22	2160	Spiolto respimat	Tiotropium + olodaterol	COPD	Pulmonology	Boehringer Ingelheim	
23	2162	Strattera	Atomoxetine	ADHD (children and adults)	Psychiatry	Eli Lilly	
23	Loose leaf	Spiolto respimat	Tiotropium + olodaterol	COPD	Pulmonology	Boehringer Ingelheim	
23	Loose leaf	Spiolto respimat	Tiotropium + olodaterol	COPD	Pulmonology	Boehringer Ingelheim	Summary of product characteristics with logo and picture.
23	2166	Relvar Ellipta	Fluticasone furoate + vilanterol	COPD + Asthma	Pulmonology	GlaxoSmithKline + Theravance	Summary of product characteristics with logo, picture, and table.
23	2166	Aduvanz	Lisdexamfetamine	ADHD (adults)	Psychiatry	Shire	Summary of product characteristics with logo.
23	2167	Aduvanz	Lisdexamfetamine	ADHD (adults)	Psychiatry	Shire	
23	2168	Relvar Ellipta	Fluticasonefuroate + vilanterol	COPD + asthma	Pulmonology	GlaxoSmithKline + Theravance	
23	2185	Panodil 665 mg	Paracetamol	Pain		GlaxoSmithKline	
23	2194	Prevenar 13	Pneumococcal vaccine	Prevention of pneumonia	Infectious disease	Pfizer	
23	2259	DuoResp Spiromax	Budesonide + formoterol	COPD + asthma	Pulmonology	TEVA Pharma	
23	2260	Brintellix	Vortioxetine	Depression	Psychiatry	Lundbeck	
24	2262	Relvar Ellipta	Fluticasone furoate + vilanterol	Asthma	Pulmonology	GlaxoSmithKline + Theravance	
24	2264	Relvar Ellipta	Fluticasone furoate + vilanterol	Asthma	Pulmonology	GlaxoSmithKline + Theravance	Summary of product characteristics with logo and table.
24	2267	Abilify Maintena	Aripiprazole	Schizophrenia	Psychiatry	Otsuka + Lundbeck	
24	2268	Anoro Ellipta	Umeclidinium + vilanterol	COPD	Pulmonology	GlaxoSmithKline + Theravance	
24	2273	Panodil 665 mg	Paracetamol	Pain		GlaxoSmithKline	
24	2292	Prevenar 13	Pneumococcal vaccine	Prevention of pneumonia	Infectious disease	Pfizer	
24	2310	Aduvanz	Lisdexamfetamine	ADHD (adults)	Psychiatry	Shire	Summary of product characteristics with logo.
24	2311	Aduvanz	Lisdexamfetamine	ADHD (adults)	Psychiatry	Shire	
24	2360	Spiolto respimat	Tiotropium + olodaterol	COPD	Pulmonology	Boehringer Ingelheim	
25	2362	Relvar Ellipta	Fluticasone furoate + vilanterol	COPD + asthma	Pulmonology	GlaxoSmithKline + Theravance	
25	2364	Relvar Ellipta	Fluticasone furoate + vilanterol	COPD + asthma	Pulmonology	GlaxoSmithKline + Theravance	Summary of product characteristics with logo and table.
25	2366	Anoro Ellipta	Umeclidinium + vilanterol	COPD	Pulmonology	GlaxoSmithKline	Summary of product characteristics with table and additional

							text.
25	2367	Prevenar 13	Pneumococcal vaccine	Prevention of pneumonia	Infectious disease	Pfizer	
25	2368	Anoro Ellipta	Umeclidinium + vilanterol	COPD	Pulmonology	GlaxoSmithKline	
25	2373	Triumeq / Tivicay	Dolutegravir + abacavir + lamivudine / dolutegravir	HIV	Infectious disease	GlaxoSmithKline + ViiV Healthcare	
25	2375	DuoResp Spiromax	Budesonide + formoterol	COPD + asthma	Pulmonology	TEVA Pharma	
25	2384	Simponi	Golimumab	Rheumatoid arthritis, psoriatic arthritis, ankylosing spondylitis, and ulcerative colitis	Rheumatology	MSD	
25	2394	Betmiga	Mirabegron	Overactive bladder	Urology	Astellas Pharma	
25	2435	Panodil 665 mg	Paracetamol	Pain		GlaxoSmithKline	
25	2460	Spiolto respimat	Tiotropium + olodaterol	COPD	Pulmonology	Boehringer Ingelheim	
26	2462	Relvar Ellipta	Fluticasone furoate + vilanterol	COPD + asthma	Pulmonology	GlaxoSmithKline + Theravance	
26	Loose leaf	Pradaxa / Praxbind	Dabigatran / idarucizumab	Prevention of thromboembolic events + antidote	Cardiology	Boehringer Ingelheim	
26	Loose leaf	Pradaxa / Praxbind	Dabigatran / idarucizumab	Prevention of thromboembolic events + antidote	Cardiology	Boehringer Ingelheim	Summary of product characteristics with logo.
26	2464	Relvar Ellipta	Fluticasone furoate + vilanterol	COPD + asthma	Pulmonology	GlaxoSmithKline + Theravance	Summary of product characteristics with logo and table.
26	2466	Praluent	Alirocumab	Hypercholesterolemia	Endocrinology	Sanofi-Aventis	
26	2556	Anoro Ellipta	Umeclidinium + vilanterol	COPD	Pulmonology	GlaxoSmithKline	Summary of product characteristics with table and additional text.
26	2560	Anoro Ellipta	Umeclidinium + vilanterol	COPD	Pulmonology	GlaxoSmithKline	

eTable 2. Medical advertisements separated into specialty and generic drug

Medical speciality	Trade name	Generic name	Drug class	Indication	Advertiser	No.	Total no. (%)
Pulmonology	Spiolto Respimat	Tiotropium + olodaterol	Anticholinergic agent + beta2-agonist	COPD	Boehringer Ingelheim	18	57 (36)
	Relvar Ellipta	Fluticasone furoate + vilanterol	Steroid + beta2-agonist	COPD + Asthma [a]	GlaxoSmithKline + Theravance	11	
	DuoResp Spiromax	Budesonide + formoterol	Steroid + beta2-agonist	COPD + asthma	TEVA	8	
	Flutiform	Fluticasone propionate + formoterol	Steroid + beta2-agonist	Asthma	Norpharma	8	
	Anoro Ellipta	Umeclidinium + vilanterol	Anticholinergic agent + beta2-agonist	COPD	GlaxoSmithKline	5	
	Duaklir Genuair	Aclidinium bromide + formoterol	Anticholinergic agent + beta2-agonist	COPD	AstraZeneca	3	
	Esbriet	Pirfenidone	Anti-inflammatory drug	Idiopathic pulmonary fibrosis	Roche	2	
	Spiriva Respimat / Striverdi Respimat	Tiotropium / olodaterol	Anticholinergic agent / beta2-agonist	COPD + asthma / COPD	Boehringer Ingelheim	1	
	Symbicort Forte Turbohaler	Budesonide + formoterol	Steroid + beta2-agonist	COPD + asthma	AstraZeneca	1	
Psychiatry	Aduvanz	Lisdexamfetamine	Central stimulant	ADHD in adults	Shire	12	32 (20)
	Brintellix	Vortioxetine	Antidepressant	Depression	Lundbeck	8	
	Abilify Maintena	Aripiprazole	Antipsychotic	Schizophrenia	Otsuka + Lundbeck	7	
	Strattera	Atomoxetine	Noradrenaline reuptake inhibitor	ADHD (children + adults)	Eli Lilly	4	
	Valdoxan	Agomelatine	Melatonine agonist	Depression	Servier	1	
Analgesics	Panodil 665 mg	Paracetamol	Analgesic and antipyretic	Pain	GlaxoSmithKline	15	17 (11)
	Norspan	Buprenorphine	Opioid	Pain	Norpharma	2	
Endocrinology	Invokana	Canagliflozin	Sodium glucose cotransporter-2 inhibitor	Diabetes mellitus type 2 (mono therapy and as add-on)	Janssen-Cilag	6	15 (9)
	Toujeo	Insulin glargine	Human insulin analogue	Diabetes mellitus (adults)	Sanofi-Aventis	4	
	Atozet	Atorvastatin + ezetimibe	Statin + cholesterol inhibitor	Hypercholesterolemia	MSD	4	
	Praluent	Alirocumab	PCSK9 antibody	Hypercholesterolemia	Sanofi-Aventis	1	
Urology	Betmiga	Mirabegron	Beta3-agonist	Overactive bladder	Astellas Pharma	5	8 (5)
	Selexid	Pivmecillinam	Antibiotic	Urinary tract infection	LEO Pharma	3	
Infectious disease	Prevenar 13	13-valent pneumococcal conjugate vaccine	Pneumococcal vaccine	Prevention of pneumonia	Pfizer	6	7 (4)
	Triumeq + Trivicay	Dolutegravir + abacavir + lamivudine/ dolutegravir	Antiviral drug	HIV	GlaxoSmithKline + ViiV	1	
Cardiology	Xarelto	Rivaroxaban	Anticoagulant	Atrial fibrillation	Bayer	4	6 (4)
	Pradaxa + Praxbind	Dabigatran + idarucizumab	Anticoagulant + antidote	Atrial fibrillation	Boehringer Ingelheim	2	
Dermatology	Acnatac	Clindamycin + tretionin	Antibiotic + retinoid	Acne vulgaris	MEDA	2	2 (1)
Rheumatology	Simponi	Golimumab	Antibody	Rheumatoid arthritis, psoriatic	MSD	5	5 (3)

				arthritis, ankylosing spondylitis, and ulcerative colitis			
**Otorhinolaryngology**	Dymista	Azelastine + fluticasone propionate	Antihistamine + steroid	Allergic rhinitis	MEDA	5	5 (3)
**Gastroenterology**	Harvoni	Ledipasvir + sofosbuvir	Antiviral drug	Hepatitis C	Gilead	2	2 (1)
**Ophthalmology**	Fucithalmic	Fusidic acic	Antibiotic	Eye infection	AMCo	1	1 (1)
**Haematology**	Zydelig	Idelalisib	Antibody	Chronic lymphatic leukaemia and follicular lymphoma	Gilead	1	1 (1)
						**Total**	158 (100)

a) Two of the 11 advertisements had asthma only as condition. COPD= chronic obstructive pulmonary disorder

eTable 3. Coincidences of drug advertisements and related scientific content

Drug (trade name)	Indication	Advertisement (Issue, page)	Related scientific content (Issue, pages)
Pirfenidon (Esbriet)	Idiopathic pulmonary fibrosis	Issue 11, page 1002	Drug mentioned in a narrative review, 'Idiopathic pulmonary fibrosis' (issue 11, pages 1060 - 1063)
Idelalisib (Zydelig)	Chronic lymphatic leukaemia and follicular lymphoma	Issue 11, page 1019	Drug mentioned in a narrative review, 'targeted treatment of chroic lymphatic leukaemia' (issue 11, pages 1053 - 1057)
Golimumab (Simponi) [a]	Rheumatoid arthritis, psoriatic arthritis, ankylosing spondylitis, and ulcerative colitis	Issue 12, page 1102	Narrative review on asthma mentioning golimumab as a new potential treatment modality (issue 12, pages 1159 - 1163) [b]
Ledipasvir / sofosbuvir (Harvoni)	Hepatitis C	Issue 12, page 1122	Narrative review on liver fibrosis (issue 12, pages 1155 – 1158)
Golimumab (Simponi)	Rheumatoid arthritis, psoriatic arthritis, ankylosing spondylitis, and ulcerative colitis	Issue 22, page 2066	Narrative review on treatment with tumour necrosis factor inhibitors, specifically mentioning golimumab (issue 22, page 2107-2108)
Dolutegravir + abacavir + lamivudine / dolutegravir (Triumeq / Tivicay)	HIV	Issue 25, page 2373	1 editorial, 1 opinion piece and 2 narrative reviews on HIV (issue 25, pages 2370 - 2371, 2397 and 2402 - 2409)
Alirocumab (Praulent)	Hypercholesterolemia	Issue 26, page 2466	Drug mentioned in a narrative review, 'Inhibition of protein PCKS9 is a promising treatment of hypercholesterolemia and prevention of cardiovascular disease', (issue 26, pages 2509-12)

[a)] In total, golimumab appeared in five advertisements during 2015, of which two advertisements (listed here) coincided with scientific content. [b)] Asthma was not listed as an indication in the summary of product characteristic**.**

Evidence for the advertised drugs

Combined beta2-agonist and steroid inhalation formulation (28 advertisements)
Treatment with long-acting beta2-agonists in combination with steroid for asthma has been controversial for many years, since two clinical trials reported that long-acting beta2-agonists increased the mortality compared to steroid only.[1] In 2011, the FDA required the conduction of five large trials (four in adults, one in children) to compare combination therapy to steroid treatment alone.[2] In 2017, the FDA reported the results from the three completed studies in adults and adolescents over 12 years (35,089 participants).[3, 4] The FDA reported an increased relative risk (RR) of asthma-related death, intubation, or hospitalisation with the combination therapy compared to steroid alone of 1.24 (CI 95%: 0.94 to 1.65) and a reduced risk of asthma exacerbations, RR of 0.84 (CI 95%: 0.78 to 0.89).[4] Based on these results the FDA removed the black box warning.[3, 4] A Cochrane review[5] from 2010 (without the FDA-requested trials) including 48 trials and 15.155 participants reported a similar reduction in asthma exacerbations (RR 0.88, 95% CI 0.78 to 0.98), a modest improvement of lung function, and there were no differences in hospital admissions or quality of life. The clinical relevance of these effects is unclear.

In chronic obstructive pulmonary disease (COPD), the benefits of combination therapy compared to beta2-agonist treatment alone are uncertain. A Cochrane review[6] could not draw conclusions regarding the added benefits of using combination therapy compared to beta2-agonist alone due to methodological deficiencies of the included trials. The review reported a decreased risk of exacerbations, but an increased risk of pneumonia with combination therapy, and no differences in mortality and hospitalisation. The effects on quality of life and lung function were small and probably clinically irrelevant.[6]

In 2016, EMA issued a referral statement that confirmed the increased risk of pneumonia with inhaled corticosteroids for patients with COPD.[7]

Combined beta2-agonist and anti-cholinergic inhalation preparations (26 advertisements)

A Cochrane review,[8] which included 10 trials and 10,894 participants, reported that combination therapy resulted in slightly improved quality of life and lung function compared to monotherapy, but the differences were not clinically relevant and there was no difference for hospitalisations or mortality. The combination treatment slightly reduced the risk of exacerbations compared to beta2-agonists alone, but not compared to anticholinergic agents alone.[8] It is therefore not clear whether combination therapy is preferable to single agent treatment for COPD.

The combination therapy was not promoted for asthma and we did not assess the evidence for this indication.

ADHD drugs (16 advertisements)

Lisdexamfetamine and atomoxetine were advertised in 2015, but not the most used attention deficit hyperactivity disorder (ADHD) drug, methylphenidate. A Cochrane review from 2018 did not identify any trials directly comparing lisdexamfetamine and methylphenidate.[9] For atomoxetine, there were seemingly no added benefits of atomoxetine over methylphenidate in the only identified head to head trial in adults.[10] Cochrane reviews on amphetamines for children and adolescents[11] and adults[9] for ADHD have concluded that the benefits compared to placebo are questionable and that the quality of the evidence is very low. A systematic review of atomoxetine for adults concluded that the drug has a poor benefit to harm balance compared to placebo.[12]

Paracetamol (15 advertisements)

The advertisements for modified-release paracetamol focused on the convenience of taking the drug three times a day instead of regular paracetamol four times a day. In 2017, EMA suspended modified-release formulations of paracetamol from the European

market because of problems with treating overdoses.[13] EMA assessed that the potential benefit of fewer daily doses did not outweighed the potential risk.

Vortioxetine (8 advertisements)

FDA and EMA approved vortioxetine for depression in 2013. A Cochrane review[14] from 2017 concluded that vortioxetine was not better than duloxetine and venlafaxine and that no direct comparisons between vortioxetine and other selective serotonin reuptake inhibitors were identified. Independent researchers published a study[15] in which they reanalysed EMA's Public Assessment Report on vortioxetine[16] and reported that vortioxetine was indeed inferior to duloxetine and venlafaxine in the pivotal head to head trials. Upon approval, EMA requested an observational post-marketing study[17] to assess several harms and FDA requested a post-marketing randomised trial to assess the longer-term effects of vortioxetine compared with placebo.[18] FDA's requested trial design[17, 18] was likely biased due to methodological limitations; all participants received vortioxetine for 16 weeks before being randomised to either continued vortioxetine or to placebo, without a taper. Such trial design may expose those participants randomised to placebo to withdrawal effects, which can likely distort the trial results.

Aripiprazole (7 advertisements)

Aripiprazole is an antipsychotic drug approved for the treatment of schizophrenia, bipolar disorder, mania, and in the United States also for depression. Aripiprazole was the best-selling drug in the United States in 2014 with a revenue of 7.5 billion Dollars.[19] According to two Cochrane reviews, it is unclear whether the drug is better or worse than other antipsychotics for the treatment of schizophrenia[20] or depression,[21] and the quality of the evidence is low and incomplete for both indications.

The advertised *Abilify Maintena* is administered as a monthly intramuscular injection. The FDA based their approval[22] of the intramuscular formulation on the ASPIRE trial,[23] which was likely biased due to withdrawal effects in the placebo group and selective inclusion of participants who tolerated the drug. All participants received aripiprazole for a minimum of 20 weeks before being randomised to either continued intramuscular aripiprazole or to placebo, without a taper (FDA review p. 16-18).[22]

EMA based their approval[24] on the ASPIRE-EU trial,[25] which compared the intramuscular formulation with regular oral tablet aripiprazole, rather than intramuscular placebo. The intramuscular and oral formulations had similar effects on the reported outcomes but EMA identified "safety concerns" of leukopenia and extrapyramidal symptoms with the intramuscular injection and requested an observational study to assess the risks.

New oral anticoagulants (6 advertisements)

Rivaroxaban and dabigatran are marketed as drugs for atrial fibrillation where regular monitoring of coagulation status (INR) is not required, as opposed to warfarin. The FDA drug approval reviews of dabigatran[26] and rivaroxaban[27] highlighted that the benefits of these drugs over warfarin were driven by poor INR control in the warfarin control groups. Independent researchers have called for reanalyses of the raw trial data or the conduction of new trials.[28]

Pneumococcal vaccine (6 advertisements)

The advertisement for the 13-valent pneumococcal vaccine stated that chronic diseases and age 50 years and older increase the risk of pneumococcal infection. A Cochrane review[29] from 2013 found no significant effects of pneumococcal vaccines (14- and 23-valent) on the risk of all-cause pneumonia or mortality in the subgroup of adults with chronic disease.[29] In 2014, the American Centers for Disease Control and Prevention (CDC) recommended the routine use of the 13-valent pneumococcal vaccine in adults aged 65 and older.[30] This recommendation was based on the CAPITA trial,[31] which randomised 84,496 participants to vaccine or placebo. After four years of follow-up, there were no differences in the risk of all-cause community acquired pneumonia, pneumonia-related or all-cause death,[31] and the number of hospital admissions were not reported.[32]

Canagliflozin (6 advertisements)

The FDA approved this sodium glucose cotransporter-2 (SGLT-2) inhibitor in 2013 for patients with type-2 diabetes who cannot be treated with metformin due to intolerance or contraindications, or as an adjunct therapy. IQWiG's assessment report[33] concluded

that canagliflozin did not have added benefits compared to glimepiride as an add-on therapy with metformin, and there was no study that directly compared canagliflozin to glimepiride as monotherapy.[33]

eTable 4. Conclusions or abstracts to support evidence categorisation

Comparison	Indication	Conclusion or relevant abstract	Our classification
Beta2-agonist + steroid inhalation *versus* steroid only	Asthma	**Cochrane review:**<br>"*There was a statistically significantly lower risk of exacerbations requiring systemic corticosteroids in patients treated with LABA and ICS (RR 0.88, 95% CI 0.78 to 0.98, 27 studies, N = 10,578) from 11.45% to 10%, with a number needed to treat of 73 (median study duration: 12 weeks). The study results were dominated by adult studies; trial data from three paediatric studies showed a trend towards increased risk of rescue oral steroids (RR 1.24, 95% CI 0.58 to 2.66) and hospital admission (RR 2.21, 95% CI 0.74 to 6.64) associated with combination therapy. Overall, there was no statistically significant difference in the risk ratios for either hospital admission (RR 1.02, 95% CI 0.67 to 1.56) or serious adverse events (RR 1.12, 95% CI 0.91 to 1.37). The combination of LABA and ICS resulted in significantly greater but modest improvement from baseline in lung function, symptoms and rescue medication use than with higher ICS dose. Despite no significant group difference in the risk of overall adverse events (RR 0.99, 95% CI 0.95 to 1.03), there was an increase in the risk of tremor (RR 1.84, 95% CI 1.20 to 2.82) and a lower risk of oral thrush (RR 0.58, 95% CI 0.40 to 0.86)) in the LABA and ICS compared to the higher ICS group. There was no significant difference in hoarseness or headache between the treatment groups. The rate of withdrawals due to poor asthma control favoured the combination of LABA and ICS (RR 0.65, 95% CI 0.51 to 0.83)*"<br>Ducharme et al. (abstract)[5]<br><br>**FDA analysis:**<br>"*[…] serious asthma-related events (a composite of hospitalization, intubation, or death), 108 of 18,006 patients (0.60%) in the inhaled-glucocorticoid group and 119 of 18,004 patients (0.66%) in the combination-therapy group had at least one composite event (relative risk in the combination-therapy group, 1.09; 95% confidence interval [CI], 0.83 to 1.43; P=0.55); 2100 patients in the inhaled-glucocorticoid group (11.7%)*	Uncertain benefits

<table>
<tr><td></td><td></td><td>and 1768 in the combination-therapy group (9.8%) had at least one asthma exacerbation (relative risk, 0.83; 95% CI, 0.78 to 0.89; P<0.001)”
Busse et al. 2018 (abstract)[3,4]</td><td></td></tr>
<tr><td>Beta2-agonist + steroid inhalation versus beta2-agonist only</td><td>COPD</td><td>Cochrane review:
“Concerns over the analysis and availability of data from the studies bring into question the superiority of ICS/LABA over LABA alone in preventing exacerbations. The effects on hospitalisations were inconsistent and require further exploration. There was moderate quality evidence of an increased risk of pneumonia with ICS/LABA. There was moderate quality evidence that treatments had similar effects on mortality. Quality of life, symptoms score, rescue medication use and FEV(1) improved more on ICS/LABA than on LABA, but the average differences were probably not clinically significant for these outcomes”.
Nannini et al. 2012 (conclusion)[6]</td><td>Uncertain evidence</td></tr>
<tr><td>Beta2-agonist + anti-cholinergic agents versus beta2-agonist only</td><td>COPD</td><td rowspan="2">Cochrane review:
“The results from this review indicated a small mean improvement in health-related quality of life and FEV1 for participants on a combination of tiotropium and LABA compared to either agent alone, and this translated into a small increase in the number of responders on combination treatment. In addition, adding tiotropium to LABA reduced exacerbations, although adding LABA to tiotropium did not. Hospital admission and mortality were not altered by adding LABA to tiotropium, although there may not be enough data. While it is possible that this is affected by higher attrition in the tiotropium group, one would expect that participants withdrawn from the study would have had less favourable outcomes; this means that the expected direction of attrition bias would be to reduce the estimated benefit of the combination treatment.”
Farne et al. 2015 (conclusion)[8]</td><td>Uncertain benefits</td></tr>
<tr><td>Beta2-agonist + anti-cholinergic agents versus anti-cholinergic agent only</td><td>COPD</td><td>Uncertain benefits</td></tr>
</table>

Atomoxetine versus extended-release methylphenidate	ADHD in adults[a]	**<u>NCT00880217 trial</u>**<br>In comparison with the active comparator extended-release methylphenidate (ER-MPH), the reported outcomes were similar (ER-MPH/atomoxetine):<br>Attrition rate (19 % versus 27 %)<br>Change from baseline on symptom scale (15.7, SD 13.3 versus 15.3, SD 14.5)<br>Any adverse event (82% versus 84%)<br>Serious adverse event (1.5% versus 0%)<br>Functional or quality of life outcomes were not reported<br><br>As reported in Weisler et al. 2010[10]	No added benefits
Lisdexamfetamine *versus* extended-release methylphenidate	ADHD in adults[a]	We did not identify relevant comparative evidence of lisdexamfetamin and methylphenidate	No evidence
Modified-release paracetamol *versus* regular paracetamol	Pain	We did not identify direct comparative evidence of modified-release and regular paracetamol.	No evidence
Vortioxetine *versus* duloxetine	Depression	**Cochrane review:**<br>*"The place of vortioxetine in the treatment of acute depression is unclear. Our analyses showed vortioxetine may be more effective than placebo in terms of response, remission and depressive symptoms, but the clinical relevance of these effects is uncertain. Furthermore, the quality of evidence to support these findings was generally low. In comparison to SNRIs, we found no advantage for vortioxetine. Vortioxetine was less effective than duloxetine, but fewer people reported adverse effects when treated with vortioxetine compared to duloxetine. However, these findings are uncertain and not well supported by evidence. A major limitation of*	No added benefits

		the current evidence is the lack of comparisons with the SSRIs, which are usually recommended as first-line treatments for acute depression. Studies with direct comparisons to SSRIs are needed to address this gap and may be supplemented by network meta-analyses to define the role of vortioxetine in the treatment of depression"[14]<br>Koesters et al. 2017 (conclusion)[14]<br><br>**<u>Reanalysis of EMA Public Assessment Report:</u>**<br>*"We carried out a meta-analysis of results of the six studies that used an active comparator (duloxetine or venlafaxine), plus a seventh more recent trial versus venlafaxine not included in the FDA and EMA reviews (Figure 2). Overall, the comparator was significantly more effective than vortioxetine at three of the four dose levels tested, including the highest dose, 20 mg, with mean differences ranging from 1.7 points (95% CI 0.5 to 2.8) at 5 mg to 3.4 points (95% CI 1.8 to 5.0) at 15 mg. Only at the 10 mg dose level were effects similar: −0.2 points (95% CI −1.5 to 1.1)"*<br>Cosgrove et al. 2016 (p. 262)[15]	
Aripiprazole intramuscular injection *versus* aripiprazole oral tablet	Schizophrenia	**<u>EMA Public Assessment Report (regarding benefits):</u>**<br>"*The proportion of responders (ie, subjects who met the stability criteria) at endpoint double blind maintenance phase was 89.8% (237/264) in the aripiprazole IM depot 400 mg/300 mg group compared with 89.4% (235/263) in the oral aripiprazole 10-30 mg group, and 75.2% (97/129) in the aripiprazole IM depot 50 mg/25 mg group. There was no significant difference in the proportion of responders between the aripiprazole IM depot 400 mg/300 mg group and the oral aripiprazole 10-30 mg group (p = 0.8750)"*<br>EPAR (p. 43)[24]<br><br>**<u>EMA Public Assessment Report (regarding harms):</u>**<br>*"The most frequently observed adverse drug reactions (ADRs) reported in ≥ 5 % of patients in two double-blind controlled clinical trials of Abilify Maintena were weight increased (9.0 %), akathisia (7.9 %), insomnia*	Uncertain evidence

		(5.8 %), and injection site pain (5.1 %). On the basis of the presented data regarding EPS (excluding akathisia) and weight gain, increasing incidences were observed over time. Given the higher frequency of EPS symptoms with the IM aripiprazole depot 400/300 mg compared to oral aripiprazole 10-30 mg and increasing incidence over treatment duration, the PRAC and CHMP agreed for a post-authorization safety study (PASS) to further investigate this known safety concern with this specific formulation"<br>EPAR (p. 62)[24]	
Dabigatran *versus* warfarin	Atrial fibrillation	**FDA Summary Review:**<br>*"Despite the apparent overall superiority of dabigatran to warfarin at the 150 mg BID dose in the population as a whole, the effect was driven entirely by patients in the warfarin group who were not as well controlled with respect to INR.*<br>*Patients whose INRs were well-controlled with warfarin had the equivalent risk of having a stroke or fatal event as those treated with dabigatran 150 mg. Thus, the superiority is really conditional, and depends on how well warfarin is used. For "real world" warfarin use in a large population, dabigatran may be superior, but this may not be true for an individual patient. It is important, therefore, not to provide dabigatran with a superiority claim to warfarin, because it would imply that even those well-treated with warfarin should be switched to dabigatran. Clearly, that is not the case."*<br>FDA Summary Review (p. 15)[26]	No added benefits
Rivaroxaban versus warfarin	Atrial fibrillation	**FDA Medical Office Review**<br>*"There is a lack of substantial evidence that rivaroxaban will have its desired effect when used as recommended in labeling. (21 CFR 314.125(b)(5)). The data from the Sponsor's Phase 3 ROCKET trial comparing rivaroxaban to warfarin are not adequate to determine whether rivaroxaban is as effective for its proposed indication in comparison to warfarin when the latter is used skillfully (e.g., TTR >~68%, near the midpoint of center based TTR in the RE-LY study, and the US median TTR of 65% in ROCKET). In order for atrial fibrillation (AFib) patients to be protected from the risk of thrombotic events, a new drug for this indication should be demonstrated to be as effective as warfarin when it is used skilfully"*	No added benefits

		FDA Medical Office Review (p. 10)[27]	
Pneumococcal vaccine *versus* placebo	Pneumococcal pneumonia	**Cochrane review:**<br>*"This meta-analysis provides evidence supporting the recommendation for PPV to prevent IPD in adults. The evidence from RCTs is less clear with respect to adults with chronic illness. This might be because of lack of effect or lack of power in the studies. The meta- analysis does not provide evidence to support the routine use of PPV to prevent all-cause pneumonia or mortality"*[29]<br><br>**CAPITA trial** (from results' section):<br>*"The vaccine did not have significant efficacy against a first episode of confirmed nonbacteremic and noninvasive pneumococcal community-acquired pneumonia when non–vaccine-type serotypes were included (P = 0.11 in the per-protocol population) or against a first episode of all-cause community-acquired pneumonia, which included nonpneumococcal as well as pneumococcal pneumonia (vaccine efficacy, 5.1%; 95% CI, −5.1 to 14.2).*<br>*The vaccine was not shown to have significant efficacy for the prevention of death from any cause. The number of deaths associated with pneumococcal disease during this study was too small to permit a meaningful analysis of the effect of the vaccine"*<br>Bonten et al. (p. 1118)[31]	Uncertain benefits
Canagliflozin *versus* glimepiride	Diabetes type 2	**IQWiG report:**<br>*"The company identified no comparative study for the assessment of canagliflozin versus the ACT for research question A and claimed no added benefit"*<br>IQWiG report (p. 2)[33]	No evidence
Metformin plus add-on canagliflozin *versus* metformin plus add-on glimepiride	Diabetes type 2	**IQWiG report:**<br>*"In the DIA3009 study, there were relevant differences between the treatment arms with regard to the specified target blood glucose levels and the therapeutic strategies determined by them: In the canagliflozin arms of the study, target blood glucose levels were not aimed at ("titration" to target levels was performed without dose changes and merely to maintain blinding) and fixed dosage was used. In the glimepiride arm, in*	Uncertain evidence

		contrast, titration was specified by an algorithm and orientated towards near-normal target levels. The substantial differences in blood-glucose lowering between treatment groups were apparently induced by the one-sided possibility of reaching a target level for glimepiride. The time course of the occurrence of the key outcomes of the DIA3009 study (hypoglycaemias) corresponded to the course of blood glucose lowering. The results of the DIA3009 study could not be used for assessing the added benefit of canagliflozin plus metformin versus the ACT specified by the G-BA because it remained unclear whether the observed effects are attributable to the drugs or to the therapeutic strategy"<br>IQWiG report (p. 21)[33]	

[a)] We decided to look only at comparisons in adults with ADHD and not also children and adolescents with ADHD, as the advertisements were targeted adults with symptoms of ADHD.

eTable 5. Post-marketing studies of advertised drugs

Studies for 9 advertised drugs, n= 20 (7 completed, 13 ongoing). Status by December 2018.

Drug (generic name)	Study ID	Status (by end 2018)	Expected /actual reporting	Requested by	Description	Clinical question
Aripiprazole intramuscular formulation	EUPAS21056	Ongoing	March 2021 (planned)	EMA	"Extrapyramidal symptoms in patients treated with Abilify Maintena®: Cohort study with a 2-year follow-up using European automated healthcare databases"	Specific harms (extrapyramidal symptoms)
Canagliflozin	EUPAS23531	Completed	Sep 2018 (actual)	EMA	"Acute Pancreatitis in Patients with Type 2 Diabetes Who are New Users of Canagliflozin as Compared with New Users of Other Antihyperglycemic Agents: A Retrospective Cohort Study Using Large Claims Databases in the United States"	Specific harms (acute pancreatitis)
	EUPAS23705	Completed	Oct 2018 (actual)	EMA	"Incidence of Diabetic Ketoacidosis among Patients with Type 2 Diabetes Mellitus Treated with SGLT2 inhibitors or Other Antihyperglycemic Agents- A Retrospective, Observational, New-User Cohort Study Using 4 Administrative Claims Databases in the US"	Specific harms (ketoacidosis)
	EUPAS27670	Ongoing	July 2019 (actual)	EMA	"Comparison of Canagliflozin vs. Alternative Antihyperglycemic Treatments on Risk of Below Knee Lower Extremity Amputation for Patients with Type 2 Diabetes Mellitus and the Subpopulation with Established Cardiovascular Disease"	Specific harms (lower limb amputations)
	NDA 204042	Ongoing	Dec 2021	FDA	"An enhanced pharmacovigilance study of ketoacidosis in patients	Specific harms

	commitment no. 1.		(planned)		treated with canagliflozin. The study will include reports of ketoacidosis or diabetic ketoacidosis for a period of 5 years, and will include assessment and analysis of spontaneous reports of ketoacidosis in patients treated with canagliflozin, with specialized follow-up to collect additional information on these cases”	(ketoacidosis)
	NDA 204042 commitment no. 3.	Ongoing	Nov 2023 (planned)	FDA	“An assessment and analysis of all foreign and domestic spontaneous reports of malignancy (pheochromocytoma, Leydig cell tumor, and renal cell carcinoma), fatal pancreatitis, hemorrhagic/necrotizing pancreatitis, severe hypersensitivity reactions (angioedema, anaphylaxis, Stevens-Johnson syndrome), photosensitivity reactions, serious hepatic abnormalities, and pregnancy in patients treated with canagliflozin. The enhanced pharmacovigilance should continue for 10 years from the date of approval for malignancies and 5 years for all other events”	Specific harms (various conditions)
Dabigatran	EUPAS7591	Completed	Dec 2016 (actual)	EMA	“Evaluation of potential off-label use of dabigatran etexilate in Europe”<br><br>“This is a descriptive, observational, multi-country European cross-sectional study of new users of dabigatran etexilate that aims to characterise on and off-label status and other medical characteristics at the time of the first captured prescription of dabigatran etexilate in each database. The study will be conducted using Cegedim Strategic Database (CSD, France), Danish National Databases (Denmark) and Clinical Practice Research Datalink (CPRD, UK).”	Clinical use
Fluticasone	EUPAS3702	Ongoing	May 2015	United	“A prospective, multicentre, non-interventional study to collect	Benefits and harms

propionate + formoterol			(planned, but data not reported)	Kingdom	further data on the safety and effectiveness of a new combination of formoterol and fluticasone in a pMDI with HFA 227 as the propellant, in subjects with mild to moderate-severe asthma.”	
	EUPAS4072	Completed	Aug 2018 (actual)	United Kingdom	“A Non interventional post authorisation study to determine the safety and effectiveness of flutiform® (Affirm Study).”	Benefits and harms
	EUPAS12330	Completed	Aug 2018 (actual)	United Kingdom	“An observational evaluation of prescribing of fixed-dose combination inhaled corticosteroid / long-acting beta2-agonist (ICS/LABA): fluticasone propionate / formoterol (FP/FOR) and adverse events in routine primary care at 18-months and 36-months post launch”	Harms
Lisdexamfetami ne	EUPAS20546	Ongoing	June 2020 (planned, but data not reported)	EMA	“Cohort Study of the Incidence of Major Cardiovascular Events in New Adult Users of Lisdexamfetamine and Remote Adult Users of Other ADHD Treatments”	Specific harms (cardiovascular events)
Rivaroxaban	EUPAS3979	Completed	June 2017 (actual)	EMA	“An Observational Post-Authorization Safety Specialist Cohort Event Monitoring Study (SCEM) to Monitor the Safety and Utilization of Rivaroxaban (Xarelto®) for the Prevention of Stroke in Patients with AF, Treatment of DVT and PE, and the Prevention of Recurrent DVT and PE in the Secondary Care Hospital Setting in England and Wales”	Harms
	EUPAS15961	Completed	Oct 2017 (actual)	EMA	“An observational post-authorization Modified Prescription-Event Monitoring safety study to monitor the safety and utilization of rivaroxaban (XARELTO®) for the prevention of stroke in patients with AF, treatment of DVT and PE, and prevention of recurrent DVT and PE following an acute DVT in the primary care setting in	Harms

					England, extended to include Acute Coronary Syndrome Patients”	
	EUPAS11299, EUPAS9895, EUPAS11141, EUPAS11145[a]	Ongoing	Oct - Dec 2020 (planned)	EMA	“A pharmacoepidemiological study of Rivaroxaban use and potential adverse outcomes in routine clinical pratice in the United Kingdom, [Sweden, the Netherlands, and Germany]”	Specific harms (bleeding events and liver disease)
Tiotropium + olodaterol	EUPAS14273	Ongoing	July 2019 (actual)	Japanese drug agency	“Post-marketing surveillance (PMS) on long-term use of tiotropium+olodaterol fixed dose combination (Tio+Olo FDC) 5/5µg in patients with chronic obstructive pulmonary disease (chronic bronchitis, emphysema) in Japan”	Long-term benefits and harms
	EUPAS14956	Ongoing	June 2022 (planned)	South Korean drug agency	“A regulatory required non interventional study to monitor the safety and effectiveness of once daily treatment of orally inhaled Vahelva Respimat (Tiotropium + Olodaterol fixed dose combination 2.5µg/2.5µg per puff (2 puffs comprise one medicinal dose)) for Korean patients with COPD (Chronic Obstructive Pulmonary Disease)”	Benefits and harms
Umeclidinium + vilanterol	EUPAS11397	Ongoing	Sep 2020 (actual)	South Korean drug agency	“An open label, multi-centre, post marketing surveillance (PMS) to monitor the safety and effectiveness of ANORO administered in Korean subjects with chronic obstructive pulmonary disease (COPD) in usual practice”	Benefits and harms
	EUPAS9868	Ongoing	Oct 2019 (actual)	Japanese drug agency	“Drug Use Investigation of ANORO ELLIPTA inhaler”	Benefits and harms

					"This investigation will be conducted to collect and evaluate information regarding the safety and efficacy of ANORO ELLIPTA under the actual post-marketing use conditions of the product."	
Vortioxetine	NDA 204447 commitment no. 6. (NCT02371980)	Ongoing	April 2020 (submitted according to site)	FDA	"A controlled trial to evaluate the longer-term (i.e., maintenance) efficacy of vortioxetine in the treatment of adults with major depressive disorder in the US. This trial must include a placebo group and several fixed doses and must utilize a randomized withdrawal design, following an adequate period of stabilization with open-label treatment of vortioxetine. Because the short-term trials appear to show that higher doses have demonstrated better treatment effects in the US population compared to the rest of the world, it is important to establish the dose-response for maintenance in the US. This trial should randomize patients on stable doses of vortioxetine to several different doses (e.g., 5 mg, 10 mg, and 20 mg) of vortioxetine (and to placebo) during the maintenance phase"	Long-term benefits and harms
	EUPAS19199	Ongoing	Dec 2021 (planned)	EMA	"A non-interventional post-authorisation safety study (PASS) of vortioxetine in Europe"	Harms

[a] We assumed that the four study numbers corresponded to the same study conducted in four different countries.

eTable 6. Post-marketing studies for comparator drugs

Studies for four comparator drugs; 10 post-marketing studies (4 completed, 6 ongoing). Status by December 2018.

Drug (generic name)	Study ID	Status (by end 2018)	Expected /actual reporting	Requested by	Description	Clinical question
Duloxetine	EUPAS20253[a]	Ongoing	Sep 2019 (actual)	United States[b]	"Observational Study to Assess Maternal and Fetal Outcomes Following Exposure to Duloxetine"	Specific harms (maternal and fetal harms)
	EUPAS20247	Completed	Sep 2017 (actual)	EMA	"Stress Urinary Incontinence and Suicidality Seen in the United Kingdom General Practice Research Database"	Specific harms (suicide)
	EUPAS7584	Completed	Sep 2013 (actual)	EMA	"We conducted a retrospective matched cohort study to evaluate the association of clinically significant hepatic injury and exposure to duloxetine, with specific definitional and methodological enhancements to focus on potential drug-related associations […]"	Specific harms (liver harms)
	EUPAS7588	Completed	June 2016 (actual)	EMA	"This study was a retrospective case-control analysis. The interaction between duloxetine and prescribed NSAIDs is described as the odds ratio (OR) for risk of UGI bleed where there is exposure to both duloxetine and prescription nonselective NSAIDs, COX-2 selective NSAIDs or prescription aspirin"	Specific harms (gastrointestinal bleeding)
	NDA 21427 commitment no. 2[a]	Ongoing	No fixed deadline	FDA	"To develop and maintain a prospective, observational pregnancy exposure registry study conducted in the United States that compares the pregnancy and fetal outcomes of women exposed to Cymbalta during pregnancy to an unexposed control population.	Specific harms (maternal and fetal harms)

					The registry will detect and record major and minor congenital anomalies, spontaneous abortions, stillbirths, elective terminations, and any serious adverse pregnancy outcomes. These events will be assessed among the enrolled women throughout the pregnancy. The events will also be assessed among infants through at least the first year of life. Annual interim reports will be submitted until FDA has acknowledged that sufficient data has been collected."	
Metformin	EUPAS5249	Completed	June 2014 (actual)	EMA	"Metformin use in renal impairment"	Specific harms (renal impairment)
Methylphenidate	EUPAS4551	Ongoing	Oct 2014 (planned, but no data reported)	EMA	"Attention Deficit Hyperactivity Disorder Drugs Use Chronic Effects: Results from the National Health Survey for Children and Adolescents (KiGGS)"<br><br>"The primary aim of this study is to examine the potential association between the use of methylphenidate and adverse outcomes using data from the German Health Interview and Examination Survey for Children and Adolescents formally conducted by the Robert Koch Institute."	Harms
	EUPAS3985	Ongoing	Oct 2015 (planned, but not data reported)	EMA	"Attention Deficit Hyperactivity Disorder Drugs Use Chronic Effects observational, open label pharmacovigilance study"<br><br>"This is a 2 year naturalistic pharmacovigilance European multicentre study investigating the long-term safety of methylphenidate in children and young people aged between 6 and 17 years."	Harms

Umeclidinium	EUPAS14947	Ongoing	Sep 2020 (actual)	South Korean drug agency	"An open label, multi-centre, post marketing surveillance (PMS) to monitor the safety and effectiveness of Incruse administered in Korean subjects with chronic obstructive pulmonary disease (COPD) in usual practice"	Benefits and harms
	EUPAS10224	Ongoing	March 2020 (actual)	Japanese drug agency	"Encruse Ellipta Drug Use Investigation"<br><br>"This investigation will be conducted to collect and evaluate information regarding the safety and efficacy of Encruse ELLIPTA under the actual post-marketing use conditions of the product"	Benefits and harms

a) The two duloxetine studies related to risk during maternity might be the same, however we include them as two separate studies. b) It is unclear why this study was registered in the European database.

eTable 7. Studies including both advertised and comparator drugs

Studies for 11 advertised and comparator drugs; 7 postmarketing studies (2 completed, 5 ongoing). Status by December 2018.

Drug (generic name)	Study ID	Status (by end 2018)	Expected /actual reporting	Requested by	Description	Clinical Question
Aclidinium and aclidinium + formoterol	EUPAS6559	Ongoing	Dec 2020 (planned)	EMA	"Aclidinium Bromide Drug Utilisation Post-Authorisation Safety Studies (DUS): Common Protocol for Aclidinium (DUS1) and Aclidinium/Formoterol Fixed-Dose Combination (DUS2)"<br><br>"DUS1/DUS2 objectives are: • To describe the characteristics and patterns of use of new users of aclidinium bromide (monotherapy or in combination with formoterol) and new users of other COPD medications. • To evaluate the potential off-label use of aclidinium bromide • To describe users of aclidinium bromide in subgroups of patients for whom there is missing information in the RMP • To establish a core cohort of new users of aclidinium bromide for the future evaluation of safety concerns described in the RMP"	Clinical use
	EUPAS13616	Ongoing	June 2023 (planned)	EMA	"Aclidinium Bromide Post-Authorisation Safety Study to Evaluate the Risk of Cardiovascular Endpoints"	Specific harms (cardiovascular and mortality)
Dabigatran and rivaroxaban, versus warfarin	EUPAS13017	Ongoing	Dec 2019 (actual)	French Regulator	"Real-life anticoagulants comparative benefit-risk in nonvalvular atrial fibrillation (NVAF) in France"	Benefits and harms

					"The research question is to compare the 1-year and long-term benefit-risk between each of direct oral anticoagulant (DOAC) (Pradaxa®, Xarelto®) vs vitamin K (VKA) for new users in nonvalvular atrial fibrillation (NVAF). The main objective is to compare the 1-year risk of major bleeding, stroke and systemic embolism (SSE) (prior named "arterial thrombotic events" as in the protocol and the study report "One-year of follow-up"), myocardial infarction (MI) and death for each DOAC (dabigatran, rivaroxaban) vs VKA in NVAF during drug exposure"	
Olodaterol and olodaterol + tiotropium	EUPAS21574	Ongoing	Sep 2020 (actual)	EMA	"Cohort study of cardiovascular events in patients with chronic obstructive pulmonary disease initiating olodaterol or other long-acting beta2-agonists"<br><br>"In the Decentralised Procedure for Striverdi Respimat, the health authorities of the European Union/European Economic Area Member States requested the conduct of a post-authorisation safety study (PASS) to gather additional data on safety in long-term use of olodaterol. The PASS will include evaluation of users of olodaterol monotherapy as well as in fixed-dose combination with tiotropium."	Specific harms (cardiovascular events)
Paracetamol (extended and regular formulation	EUPAS33976	Completed	June 2018 (actual)	EMA	"Prenatal exposure to paracetamol and the risk of urogenital system disorders or neurodevelopmental disorders in offspring: a systematic review of observational studies"	Specific harms (urogenital and neurodevelopmental disorders)

Rivaroxaban (dabigatran and warfarin)	EUPAS14567	Completed	Nov 2018 (actual)	French regulator	“Benefit-risk of arterial thrombotic prevention with rivaroxaban for atrial fibrillation in daily clinical practice”<br><br>“The research question is to assess the one-year and two-year benefit-risk of rivaroxaban for stroke prevention in atrial fibrillation (SPAF) compared to vitamin K antagonists (VKA) and dabigatran among new anticoagulant users. The main objective is to compare the one-year and two-year risk of the following individual outcomes: stroke and systemic embolism (SSE), major bleeding and death, between new users of anticoagulant for SPAF during drug exposure: rivaroxaban versus VKA, and rivaroxaban versus dabigatran (standard and reduced doses)”	Benefits and harms
Umeclidinium and umeclidinium/ vilanterol	EUPAS10316	Ongoing	Aug 2021 (planned)	EMA	“Post-authorisation Safety (PAS) Observational Cohort Study to Quantify the Incidence and Comparative Safety of Selected Cardiovascular and Cerebrovascular Events in COPD Patients Using Inhaled UMEC/VI Combination or Inhaled UMEC versus Tiotropium (Study 201038)”	Specific harms (cardiovascular and cerebrovascular events)

eTable 8. Search overview for comparative evidence

Comparison	Indication	Cochrane Library	IQWiG	FDA	EMA
Beta2-agonist + steroid inhalation *versus* steroid alone	Asthma	*Ducharme et al, 2010[34]	Not searched	Not searched	Not searched
Beta2-agonist + steroid inhalation *versus* beta2-agonist only	COPD	Nannini et al, 2012[6]	Not searched	Not searched	Not searched
Beta2-agonist + anti-cholinergic agents *versus* beta2-agonist only	COPD	Farne et al, 2015[8]	Not searched	Not searched	Not searched
Beta2-agonist + anti-cholinergic agents *versus* anti-cholinergic agent only	COPD	Farne et al, 2015[8]	Not searched	Not searched	Not searched
Lis-dexamfetamine versus methylphenidate	ADHD	Castells et al, 2018[9]	Not searched	Not searched	Not searched
Atomoxetine versus methylphenidate	ADHD	*Boesen et al, 2017[35]	No relevant hits	No relevant hits	No relevant hits
Modified-release paracetamol *versus* regular paracetamol	Pain	No relevant hits*	No relevant hits	No relevant hits	No relevant hits
Vortioxetine *versus* duloxetine	Depression	*Koesters et al, 2013[14]	Not searched	Not searched	Not searched
Aripiprazole intramuscular injection *versus*	Schizophrenia	No relevant hits	No relevant hits	No relevant hits	EMA Public Assessment Report[24]

aripiprazole oral tablet					
Dabigatran *versus* warfarin	Atrial fibrillation	*Salazar et al, 2014[36]	No relevant hits	FDA Medical Office Review[26]	Not searched
Rivaroxaban *versus* warfarin	Atrial fibrillation	*Bruins Slot KM et al, 2018[37]	No relevant hits	FDA Medical Office Review[27]	Not searched
Pneumococcal vaccine *versus* placebo	Pneumococcal infection	*Moberley et al, 2013[29]	Not searched	Not searched	Not searched
Canagliflozin *versus* glimepiride	Diabetes type 2	No relevant hits*	IQWiG Report[33]	Not searched	Not searched
Metformin plus add-on canagliflozin *versus* metformin plus add-on glimepiride	Diabetes type 2	No relevant hits*	IQWiG Report[33]	Not searched	Not searched

Comments to the search strategy marked with an asterisk (*) are explained in **eTable 8**.

Detailed search results

We searched the databases in a hierarchal order of Cochrane Library, IQWiG's project database, Drugs@FDA, and EMA Medicines. Comments and caveats to our search are described in **eTable 8**.

Beta2-agonist + steroid inhalation *versus* steroid alone

Cochrane Library
Search term: We used the tags: "Lungs and airways", "Asthma (chronic)", "Combination/additive treatment".
Hits: 25

Beta2-agonist + anti-cholinergic agents *versus* beta2-agonist only

Cochrane Library
Search term: We used the tags: "Lungs and airways", "Chronic Obstructive Pulmonary Disorder (stable)", "Combination/additive treatment".
Hits: 14

Lis-dexamfetamine *versus* methylphenidate

Cochrane Library
Search term: "lis-dexamfetamine and amphetamine"
Hits: 3

Atomoxetine versus methylphenidate

Cochrane Library
Search term: "atomoxetine"
Hits: 7

IQWiG

Search term: atomoxetine

Hits: 0

FDA

Search term: atomoxetine

Hits: 2

EMA

Search terms: “atomoxetine”

Hits: 7

Modified-release paracetamol *versus* regular paracetamol

Cochrane Library

Search term: “Paracetamol”.

Hits: 119

IQWiG

Search term: “Paracetamol”

Hits: 0

Drugs@FDA

Search term: “Acetaminophen”

Hits: 106

EMA medicines

Search term: “Paracetamol”

Hits: 189

Vortioxetine *versus* duloxetine

Cochrane Library

Search term: “vortioxetine”

Hits: 1

Aripiprazole intramuscular injection *versus* oral tablet aripiprazole

Cochrane Library

Search term: “aripiprazole”

Hits: 26

IQWiG

Search term: “aripiprazole”

Hits: 0

FDA

Search term: “aripiprazole”

Hits: 6

EMA

Search term: “aripiprazole”, EPAR

Hits: 17

Dabigatran *versus* warfarin

Cochrane Library

Search term: “dabigatran”

Hits: 8

IQWiG

Search term: dabigatran

Hits: 0

Drugs@FDA

Search term: dabigatran

Hits: 2

Rivaroxaban *versus* warfarin

Cochrane Library

Search term: “rivaroxaban”

Hits: 10

IQWiG

Search term: “rivaroxaban”

Hits: 0

Drugs@FDA

Search term: “rivaroxaban”

Hits: 1

Pneumococcal vaccine *versus* placebo

Cochrane Library

Search term: “Pneumococcal”

Hits: 16

Canagliflozin *versus* glimepiride

Cochrane Library

Search term: “Canagliflozin”

Hits: 1

IQWiG

Search term: “Canagliflozin

Hits: 1

Metformin + canagliflozin add-on *versus* metformin + glimepiride add-on

Cochrane Library

Search term: “Canagliflozin”

Hits: 1

IQWiG

Search term: “Canagliflozin

Hits: 1

eTable 9. Comments on the search strategy

Advertised drug	Comments
Beta2-agonist + steroid inhalation *versus* steroid alone	There were two Cochrane reviews comparing combination therapy to either same dose steroid or higher dose steroid. We chose the review with higher dose steroid.<br><br>We identified an FDA Safety Communication regarding an FDA analysis,[3] and later a published report of the analysis,[4] of four randomised clinical trials that were conducted after publication of the Cochrane review.[5] The FDA analysis[3, 4] specifically addressed our research question so we decided to also included it in our evidence assessment. It did not change our evidence categorisation.
Atomoxetine *versus* methylphenidate for ADHD	We were not able to locate the FDA Medical Office Review for the approval of atomoxetine for adults, presumably because it was an extension to the original approval of atomoxetine for children and adolescents. A narrative review describing the approval process for use in adults highlighted that atomoxetine was not tested against active comparators in the pivotal trials.[38] In Europe, atomoxetine was approved by the German drug regulators and not centrally by EMA.<br><br>We are the author group of the extended-release methylphenidate Cochrane review[35] and through this work we had knowledge of the only published trial[10] that compared atomoxetine and extended-release methylphenidate.
Modified release paracetamol *versus* regular paracetamol	We did not find any review that assessed modified versus regular paracetamol. We identified a Cochrane overview of reviews of analgesics.[39] In this overview, one review on paracetamol was included[40] but the included studies in this review were single-dose studies and not relevant.<br><br>None of the hits in the FDA database were relevant. None of the hits labelled "extended-release" were manufactured by GSK, the sponsor of the advertisement.
Vortioxetine *versus* duloxetine and venlafaxine	In addition to the Cochrane review, we became aware of a reanalysis[15] of vortioxetine's EMA Public Assessment Report and decided to include it. It did not change the evidence categorisation based on the Cochrane review.[14]
Aripiprazole intramuscular injection versus oral tablet aripprazole	The FDA review[22] relied on evidence from the ASPIRE trial,[23] aripiprazole intramuscular injection versus inert placebo. We therefore also searched the EMA medicines database of Public Assessment Reports. In EMA's aripiprazole Public Assessment Report[24] we identified the ASPIRE-EU trial

	that included a direct comparison with aripiprazole oral tablet.
Pneumococcal vaccine *versus* placebo	We became aware of the CAPITA[31] trial indirectly through one of the references on the medical advertisement (the CAPITA trial was not directly referenced in the advertisement). The CAPITA trial directly assessed our research question and since the included Cochrane review[29] was published before this trial, we decided to include it in our evidence assessment. It did not change our categorisation of the evidence.
Dabigatran *versus* warfarin	Important criticism of the published Cochrane review[36] was published in October 2014 in the Cochrane Library, which (as of November 2019) had not been addressed by the review authors. We therefore decided to continue the search for available evidence.
Rivaroxaban *versus* warfarin	Important criticism had been raised for the previous version of this Cochrane review, published in 2014, including concerns of the impact of time in therapeutic range (TTR) of the warfarin control group. In the updated review from 2018[37] some of these issues had been addressed. However, we noted that the Cochrane review only addressed the critical issue of TTR of warfarin in a subgroup analysis dichotomising the participants as being in TTR above or below 60 % of the time (specified in the "**Subgroup analysis and investigation of heterogeneity"** section). We found this approach inadequate to make a fair comparison of rivaroxaban versus warfarin and we therefore decided to continue the search for available evidence.
Canagliflozin *versus* glimepiride	The Cochrane review[41] did not include any trials with canagliflozin, so we continued to search for evidence.
Metformin plus add-on canagliflozin *versus* metformin plus add-on glimepiride	The Cochrane review[41] did not include any trials with canagliflozin, so we continued to search for evidence.

eReferences

1) Chowdhury BA, Dal Pan G. The FDA and safe use of long-acting beta-agonists in the treatment of asthma. *N Engl J Med.* 2010;362(13):1169-71.
2) FDA Drug Safety Communication: FDA requires post-market safety trials for Long-Acting Beta-Agonists (LABAs). 15 April 2011. Available from: https://www.fda.gov/Drugs/DrugSafety/ucm251512.htm (accessed 28 January 2019).
3) FDA Drug Safety Communications: FDA review finds no significant increase in risk of serious asthma outcomes with long-acting beta agonists (LABAs) used in combination with inhaled corticosteroids (ICS). 20 December 2017. Available from: https://www.fda.gov/media/109953/download (Accessed 28 January 2019).
4) Busse WW, Bateman ED, Caplan AL, et al. Combined analysis of asthma safety trials of long-acting beta2-agonist. *N Engl J Med.* 2018; 378(26):2497-2505.
5) Ducharme FM, Chroinin MN, Greenstone I, Lasserson TJ. Addition of long - acting beta2 - agonists to inhaled steroids versus higher dose inhaled steroids in adults and children with persistent asthma. *Cochrane Database Syst Rev.* 2010;(4):CD005533.
6) Nannini JL, Lasserson TJ, Poole P. Combined corticosteroid and long-acting $beta_2$-agonist in one inhaler versus long-acting $beta_2$-agonists for chronic obstructive pulmonary disease. *Cochrane Database Syst Rev.* 2012;(9):CD006829.
7) European Medicines Agency. EMA completes review of inhaled corticosteroids for chronic obstructive pulmonary disease. 14 July 2016. Available from: https://www.ema.europa.eu/en/medicines/human/referrals/inhaled-corticosteroids-containing-medicinal-products-indicated-treatment-chronic-obstructive (assessed 28 January 2019).
8) Farne HA, Cates CJ. Long-acting $beta_2$-agonist in addition to tiotropium versus either tiotropium or long-acting $beta_2$-agonist alone for chronic obstructive pulmonary disease. *Cochrane Database Syst Rev.* 2015;(10):CD008989.
9) Castells X, Blanco-Silvente L, Cunill R. Amphetamines for attention deficit hyperactivity disorder (ADHD) in adults. *Cochrane Database Syst Rev.* 2018;(8):CD007813.
10) Weisler RH, Pandina GJ, Daly EJ, Cooper K, Gassmann-Mayer C. Randomized clinical study of a histamine H3 receptor antagonist for the treatment of adults with attention-deficit hyperactivity disorder. *CNS Drugs.* 2012;26(5):421-34.

11) Punja S, Shamseer L, Hartling L, et al. Amphetamines for attention deficit hyperactivity disorder (ADHD) in children and adolescents. *Cochrane Database Syst Rev.* 2016;(2):CD009996.
12) Cunill R, Castells X, Tobias A, Capellà D. Atomoxetine for attention deficit hyperactivity disorder in the adulthood: a meta-analysis and meta-regression. *Pharmacoepidemiol Drug Saf.* 2013;22(9):961-9.
13) European Medicines Agency. Modified-release paracetamol-containing products to be suspended from EU market. 15 December 2017. Available from: http://www.ema.europa.eu/ema/index.jsp?curl=pages/news_and_events/news/2017/12/news_detail_002876.jsp&mid=WC0b01ac058004d5c1 (accessed 16 January 2019).
14) Koesters M, Ostuzzi G, Guaiana G, Breilmann J, Barbui C. Vortioxetine for depression in adults. *Cochrane Database Syst Rev.* 2017;(7):CD011520.
15) Cosgrove L, Vannoy S, Mintzes B, Shaughnessy AF. Under the influence: The interplay among industry, publishing, and drug regulation. *Account Res.* 2016;23(5):257-79.
16) European Medicines Agency. Vortioxetine public assessment report. 24 October 2014. Available from: https://www.ema.europa.eu/en/documents/assessment-report/brintellix-epar-public-assessment-report_en.pdf (accessed 16 January 2019).
17) Center for drug evaluation and research. 204447Orig1s000. Approval letter. 30 September 2013. Available from: https://www.accessdata.fda.gov/drugsatfda_docs/nda/2013/204447Orig1s000Approv.pdf (accessed 28 January 2019).
18) Clinicaltrials.gov. NCT02371980. Available from: https://clinicaltrials.gov/ct2/show/NCT02371980 (accessed 28 January 2019).
19) Brown T. 100 most prescribed, best-selling branded drugs through September. 3 November 2014. Available from: http://www.medscape.com/viewarticle/834273#vp_4 (Accessed 9 October 2016).
20) Khanna P, Suo T, Komossa K, et al. Aripiprazole versus other atypical antipsychotics for schizophrenia. *Cochrane Database Syst Rev.* 2014;(1):CD006569.
21) Komossa K, Depping AM, Gaudchau A, et al. Second-generation antipsychotics for major depressive disorder and dysthymia. *Cochrane Database Syst Rev.* 2010;(12):CD008121.
22) Center for drug evaluation and research. NDA 202971Orig1s000. Medical review. 28 September 2012. Available from:

https://www.accessdata.fda.gov/drugsatfda_docs/nda/2013/202971Orig1s000MedR.pdf (accessed 26 January 2019).

23) NCT00705783. Intramuscular Depot Formulation of Aripiprazole as Maintenance Treatment in Patients With Schizophrenia (ASPIRE). Available from: https://clinicaltrials.gov/ct2/show/NCT00705783 (accessed 5 Nov 2019).

24) European Medicines Agency. EMA/737723/2013. Assessment report Abilify Maintena. 19 September 2013. Available from: https://www.ema.europa.eu/documents/assessment-report/abilify-maintena-epar-public-assessment-report_en.pdf (accessed 21 January 2019).

25) NCT00706654. Intramuscular Depot Formulation of Aripiprazole as Maintenance Treatment in Patients With Schizophrenia (ASPIRE). Available from: https://clinicaltrials.gov/ct2/show/NCT00706654 (accessed 5 Nov 2019).

26) Center for drug evaluation and research. NDA 22-152. Summary review. 19 Oct 2010. Available from: https://www.accessdata.fda.gov/drugsatfda_docs/nda/2010/022512Orig1s000SumR.pdf (accessed 28 January 2019).

27) Center for drug evaluation and research. NDA 22-439. Medical office review. 10 Aug 2011. Available from: https://www.accessdata.fda.gov/drugsatfda_docs/nda/2011/202439Orig1s000MedR.pdf (Accessed 28 January 2019).

28) Mahtani KR, Heneghan C. Novel oral anticoagulants for atrial fibrillation. *BMJ. 2016;*354:i5187.

29) Moberley S, Holden J, Tatham DP, Andrews RM. Vaccines for preventing pneumococcal infection in adults. *Cochrane Database Syst Rev.* 2013;(1):CD000422.

30) Tomczyk S, Bennett NM, Stoecker C, et al. Use of 13-valent pneumococcal conjugate vaccine and 23-valent pneumococcal polysaccharide vaccine among adults aged ≥65 years: recommendations of the advisory committee on immunization practices (ACIP). *MMWR Morb Mortal Wkly Rep.* 2014;63(37):822–5.

31) Bonten MJ, Huijts SM, Bolkenbaas M, et al. Polysaccharide conjugate vaccine against pneumococcal pneumonia in adults. *N Engl J Med.* 2015;372(12):1114-25.

32) Swartz AW. Vaccine against pneumococcal pneumonia in adults. *N Engl J Med.* 2015;373(1):91-2.

33) IQWiG Reports – Commission No. A14-12. Canagliflozin – Benefit assessment according to §35a Social Code Book V. 12 June 2014. Available from:

https://www.iqwig.de/en/projects-results/projects/drug-assessment/a14-12-canagliflozin-benefit-assessment-according-to-35a-social-code-book-v-dossier-assessment.6098.html (Accessed 28 January 2019).

34) Ducharme FM, Chroinin MN, Greenstone I, Lasserson TJ. Addition of long-acting beta2-agonists to inhaled steroids versus higher dose inhaled steroids in adults and children with persistent asthma. *Cochrane Syst Database Rev* 2010;14: CD005533.
35) Boesen K, Danborg PB, Gøtzsche PC, Jørgensen KJ. Extended-release methylphenidate for attention deficit hyperactivity disorder (ADHD) in adults (protocol). *Cochrane Database Syst Rev* 2017;11:CD012857.
36) Salazar CA, del Aguila D, Cordova EG. Direct thrombin inhibitors versus vitamin K antagonists for preventing cerebral or systemic embolism in people with non-valvular atrial fibrillation. *Cochrane Database Syst Rev* 2014;3:CD009893.
37) Bruins Slot KM, Berge E. Factor Xa inhibitors versus vitamin K antagonists for preventing cerebral or systemic embolism in patients with atrial fibrillation. *Cochrane Database Syst Rev* 2018;3: CD008980.
38) Simpson D, Plosker GL. Atomoxetine. A review of its use in adults with attention deficit hyperactivity disorder. *Drugs* 2004; 64:205-222.
39) Moore RA, Wiffen PJ, Derry S, Maguire T, Roy YM, Tyrrell L. Non-prescription (OTC) oral analgesics for acute pain - an overview of Cochrane reviews. *Cochrane Database Syst Rev* 2015;11: CD010794.
40) Toms L, McQuay HJ, Derry S, Moore RA. Single dose oral paracetamol (acetaminophen) for postoperative pain in adults. *Cochrane Database Syst Rev* 2008;4:CD004602.
41) Hemmingsen B, Krogh J, Metzendorf MI, Richter B. Sodium-glucose cotransporter (SGLT) 2 inhibitors for prevention or delay of type 2 diabetes mellitus and its associated complications in people at risk for the development of type 2 diabetes mellitus. *Cochrane Database Syst Rev* 2016; 4:CD012106.
